# From molecular correction to functional rescue: delivery, tissue state, and immunobiology as the principal constraints on dystrophin-restoring therapy in Duchenne muscular dystrophy

**DOI:** 10.3389/fphar.2026.1930837

**Published:** 2026-09-22

**Authors:** Ke Li, Huan Qi, Yaoyao Xin, Lun Qin, Yulong Cheng, Xin Yao

**Affiliations:** 1 Henan General Hospital, Zhengzhou, Henan, China; 2 Zhengzhou Central Hospital Affiliated to Zhengzhou University, Zhengzhou, Henan, China; 3 Peking University First Hospital, Beijing, China; 4 Nancao Village Public Health Center, Zhengzhou, Henan, China; 5 The Third Affiliated Hospital, Henan University of Chinese Medicine, Zhengzhou, Henan, China

**Keywords:** cardiomyopathy, Duchenne muscular dystrophy, dystrophin restoration, genome editing, immunogenicity

## Abstract

Duchenne muscular dystrophy (DMD) arises from thousands of distinct mutations at a single X-linked locus that converge on loss of functional dystrophin, and this convergence on one molecular endpoint—rather than any genetic uniformity—has long made the disease appear a candidate for straightforward molecular correction. Three decades of work indicate that the difficulty lies less in restoring dystrophin than in restoring it adequately along four axes: amount, retained domains, breadth of coverage across skeletal muscle, diaphragm, heart and brain—the constraint being extent of coverage rather than correctness of sarcolemmal localization, which these platforms achieve reliably—and persistence in regenerating tissue. We argue that DMD is best understood as a systemic delivery problem superimposed on a progressive regenerative disorder, in which cargo design, biodistribution, cardiac coverage, immune response, disease stage and endpoint design jointly determine clinical benefit. AAV-microdystrophin has shown that body-wide expression is achievable, but delivers a truncated protein from an episomal genome at a dose whose upper bound was set by toxicity in other indications and whose minimum effective value was established in mdx mice rather than titrated in patients, leaving the therapeutic window better defined at its toxic than at its lower boundary. Exon skipping establishes that low-magnitude restoration is not trivial; genome editing addresses durability but adds integration risk; non-viral carriers permit redosing yet lack the potency required for a compartment comprising a third of body mass. Immunobiology constrains the field at several sequential levels, each now partially modifiable. Because the immunological burden of body-wide delivery can itself limit benefit, safety and efficacy are not fully separable, and progress is better judged by corrected muscle mass relative to immunological burden than by peak biopsy expression.

## 1 Introduction

DMD has attracted sustained therapeutic interest because its genetic basis is comparatively well defined. It is not a syndrome of uncertain origin or a trait shaped by many small-effect loci; it is a monogenic disease caused by the absence of functional dystrophin—although, as set out below, the mutations at that locus are numerous and their clinical consequences are not uniform. After the *DMD* gene was cloned and characterized in the late 1980s (Kunkel et al., 1986; Koenig et al., 1987), it was reasonable to expect that restoring the missing gene or its product would substantially correct the disease, and this expectation shaped subsequent development across platforms (Lee et al., 1991; Chamberlain, 1992).

Experience has clarified the limits of that expectation, and it is worth being precise about what is, and is not, simple here. DMD is genetically unitary rather than genetically simple: essentially all cases converge on loss of functional full-length dystrophin (Dp427m) encoded at a single X-linked locus, and it is this convergence on a shared molecular endpoint—not any simplicity of genotype—that makes one therapeutic objective definable across patients. The mutation spectrum itself is anything but simple. More than 7,000 distinct pathogenic variants have been catalogued, including large deletions and duplications, small insertions and deletions, nonsense, splice-site and deep-intronic changes, and this spectrum is a major source of clinical heterogeneity rather than a neutral background to it Bladen et al. (2015). Whether a variant preserves the reading frame largely determines whether the phenotype is Duchenne or Becker, and well-documented exceptions to the reading-frame rule show that this mapping is itself imperfect (Monaco et al., 1988; Aartsma-Rus et al., 2006; Deburgrave et al., 2007); even very low levels of residual dystrophin arising from partial splicing or revertant events are associated with measurably milder disease (de Feraudy et al., 2021); the position of the mutation relative to the internal promoters of the locus determines which shorter isoforms are lost and thereby a substantial part of the cognitive and behavioural phenotype (Pane et al., 2012; Chamova et al., 2013; Ricotti et al., 2016); and deletion boundaries determine which epitopes encoded by a therapeutic construct a given patient has never been immunologically tolerized to, and hence the risk of transgene-directed immunity ((Bönnemann et al., 2023); Section 8.2). Genotype in DMD is therefore a determinant of phenotype, of trial eligibility, and of therapeutic risk. The sense in which we use “simple” throughout this review is thus deliberately restricted: it refers to the existence of a single, well-defined molecular endpoint—restoration of functional dystrophin—and not to genetic or clinical uniformity. Even so defined, that endpoint proves therapeutically complex, largely because dystrophin and its isoforms are required across an unusually large and mechanically active tissue compartment: hundreds of skeletal muscles, the diaphragm, and the heart (Lopez Perez and Weisleder, 2026), as well as the central nervous system (Section 2). The target is therefore not a discrete organ but a distributed system, and this is a central reason why conceptual clarity has not translated into clinical simplicity.

The questions the field asks have shifted accordingly. Whether dystrophin restoration is achievable has been demonstrated repeatedly—in transgenic animals, in antisense-treated muscle, in AAV-transduced tissue, and, in preclinical settings, through genome editing (Duan et al., 2021). The more difficult and now more relevant question is what form of restoration is sufficient to change the natural history of the disease in muscle that has already diverged anatomically, immunologically, and functionally from normal tissue. The relevant limitation is not that the restored protein is mislocalized. Where dystrophin or microdystrophin is expressed following AAV gene transfer or antisense-mediated exon skipping, it is consistently reported at the sarcolemma in the expected subcellular position, and it recruits dystrophin-associated glycoprotein complex components as predicted (Cirak et al., 2011; Mendell et al., 2013; Mendell et al., 2020; Zaidman et al., 2023). The variable is coverage rather than localization: what proportion of myofibres within a muscle, what proportion of myonuclei within a fibre, and which muscles and organs across the body have been reached. A protein can therefore be correctly localized in every fibre in which it is expressed and still be present in too few fibres, too few myonuclei per fibre, or too few of the compartments that determine clinical outcome. A vector can transduce skeletal muscle while performing poorly in the heart; biopsy-level expression can be substantial without producing a clear short-term functional signal; and a biologically active therapy can still fail to demonstrate benefit for reasons unrelated to its molecular activity: a transgene-directed T-cell response may eliminate the very fibres that have been corrected; treatment given after fibro-fatty replacement is advanced may act on too few surviving myofibres to change function; and a 48- to 52-week primary endpoint may be too short to detect a reduction in the rate of decline rather than an absolute gain in strength.

This review is organized around a single proposition: the central problem in DMD is not simply to restore dystrophin, but to restore dystrophin that is biologically adequate in amount, structure, distribution and persistence (Figure 1), and to do so at an appropriate disease stage, in the relevant tissues, at a sufficient density, and without provoking systemic harm that offsets the benefit (Figure 2). Viewed this way, several features of the field become easier to interpret. The conditional or accelerated regulatory status of AAV-microdystrophin appears neither as unqualified success nor as failure, but as the first strategy able to place a dystrophin-like protein into striated muscle body-wide—achieved by accepting a truncated transgene, an episomal genome, and a vector dose near the ceiling of immunological tolerance (Oskoui et al., 2025; Pascual-Morena et al., 2025). The variable clinical results of exon-skipping drugs are consistent with the observation that low-level restoration can be mechanistically genuine yet functionally modest (Roshmi and Yokota, 2023; Harper et al., 2024). Interest in genome editing reflects its potential to address the durability limitations of episomal gene transfer rather than any demonstrated solution to DMD (Singh et al., 2024). The recent safety events—hepatotoxicity, complement activation, thrombotic microangiopathy, and transgene-directed myositis—are better read as evidence that immune biology constrains this class of therapy at multiple levels than as isolated toxicities (Bönnemann et al., 2023; Potter et al., 2025).

**FIGURE 1:**
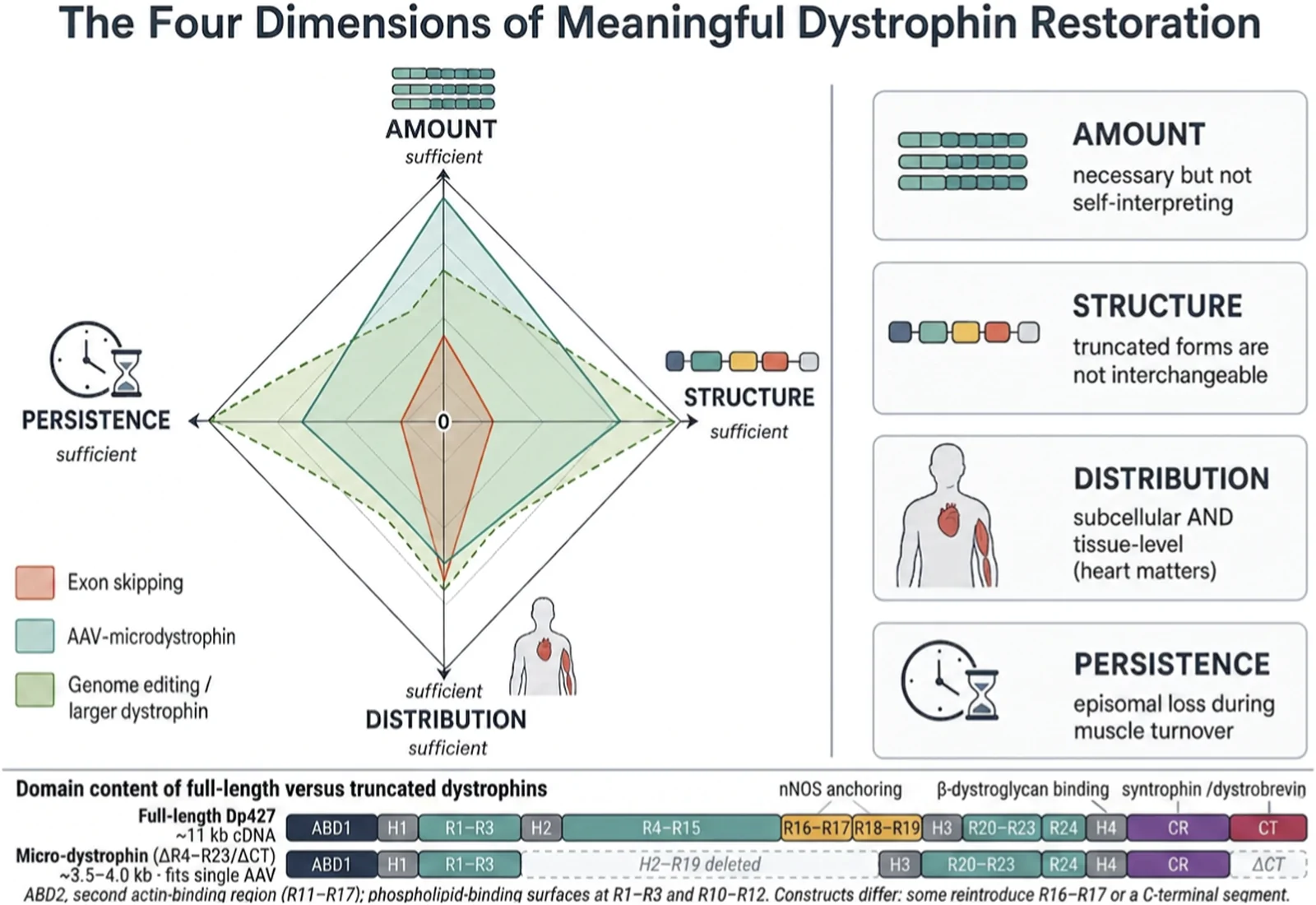
Meaningful dystrophin restoration as a four-dimensional state. Restoration is often discussed as a single event (“dystrophin present vs. absent”), but adequacy depends jointly on four partly independent variables: the amount of protein, its structural form (which domains are retained; lower panel), its coverage—meaning the fraction of myonuclei per fibre, the fraction of fibres per muscle, and the tissue compartments reached, including the myocardium; note that correct sarcolemmal localization is achieved by all of these platforms wherever expression occurs and is therefore not the discriminating variable - and the persistence of expression in regenerating muscle. Representative platforms occupy different, incomplete regions of this space—antisense exon skipping tends toward low amount and modest structure, AAV-microdystrophin toward higher but structurally incomplete and often mosaic expression, and genome editing toward durable native-locus correction with as-yet-unproven distribution (dashed). Delivery platform is orthogonal to this space: non-viral carriers (LNP, polymeric and inorganic nanoparticles) do not alter which region of the space a cargo occupies, but uniquely allow the persistence dimension to be satisfied by a permanent edit rather than by a persistent vector, while permitting repeat administration. No current approach satisfies all four dimensions simultaneously, which is why molecular activity and clinical benefit are not linearly related. Lower panel. Domain content of full-length Dp427 compared with a representative mini-dystrophin (ΔH2–R19) and a representative single-AAV micro-dystrophin (ΔR4–R23/ΔCT). Micro-dystrophins retain the N-terminal actin-binding domain, hinges 1, 3 and 4, repeats R1–R3, R24 and the cysteine-rich domain, but omit most of the rod—including the nNOS-anchoring repeats R16–R17, the second actin-binding region (R11–R17) and part of the phospholipid-binding surface (R10–R12)—together with the C-terminal syntrophin/dystrobrevin-binding domain; individual constructs differ in whether R16–R17 or a C-terminal segment is reintroduced. AAV-delivered U7 snRNA is plotted separately because, in exon 2 duplication genotypes, it occupies the maximal position on the structural axis—full-length wild-type 427-kDa dystrophin with all domains retained—while remaining constrained on the persistence axis: an inverse profile to AAV-microdystrophin.

**FIGURE 2:**
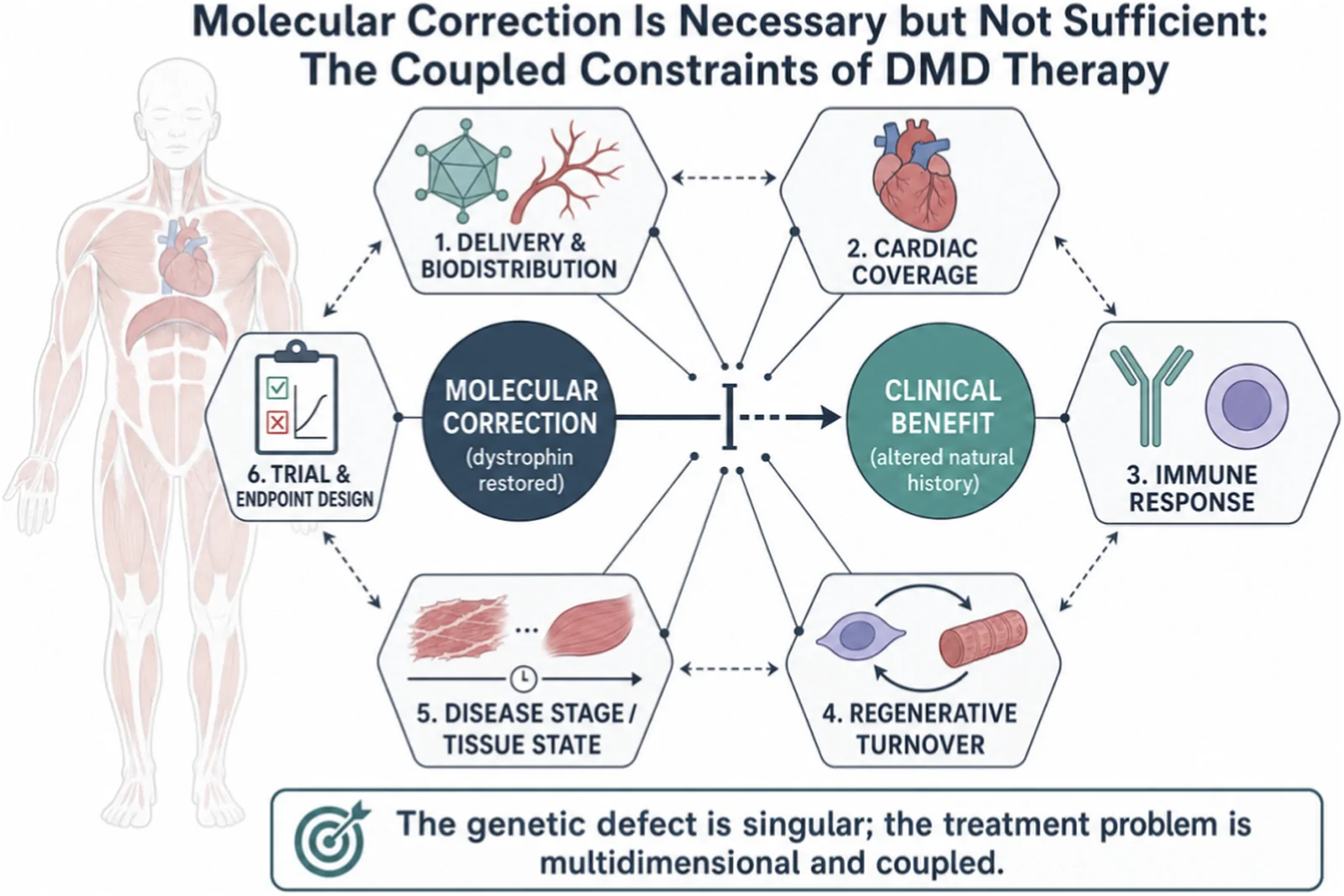
DMD reframed as a distributed delivery problem superimposed on a progressive regenerative disorder. Dystrophin and its shorter isoforms are required across a large, distributed set of compartments (background silhouette: skeletal muscles, diaphragm, heart, and central nervous system), which differ not only in accessibility but in the nature of the deficit—mechanical and degenerative in striated muscle, largely developmental and synaptic in brain. Consequently, restoring dystrophin at the molecular level (left) does not translate directly into clinical benefit (right). The conversion is gated and modulated by six interdependent constraints—delivery/biodistribution, cardiac coverage, immune response, regenerative turnover, disease stage/tissue state, and trial-and-endpoint design. A seventh constraint, CNS coverage, is shown as a separate node because it is currently limited less by biodistribution than by construct design: the muscle-restricted promoters used clinically preclude neuronal expression by intention (Section 9.2). Dashed connectors indicate that these constraints interact rather than act independently (for example, the immunological burden required for body-wide delivery can itself limit benefit). This coupling is the central organizing premise of the review. Cardiac coverage is shown as a distinct constraint because, unlike skeletal muscle, it cannot be verified by biopsy; it is instead inferred from non-invasive surrogates (CMR late gadolinium enhancement, native T1/T2 mapping, ECV, strain, and hs-cTnI) that report myocardial tissue state rather than vector delivery, and that change over years rather than months.

A second premise underlies the review. DMD is not only a disease of membrane fragility; it is also a disease of progressive regenerative failure. Myofibres are lost progressively and are incompletely replaced, satellite cells show impaired asymmetric division and reduced expansion capacity, and fibrous and adipose tissue accumulate. The muscle that a vector enters at 4 years of age therefore differs from the same muscle at 10 years in three measurable respects: the number of transducible myofibres, the proportion of muscle volume that is non-contractile, and the regenerative reserve available to maintain the corrected state (Dumont et al., 2015; Kodippili and Rudnicki, 2023). The same construct at the same dose is therefore not the same intervention at 4 years and at 12 years: the later infusion encounters fewer viable myofibres, a larger non-contractile compartment, a partially depleted satellite-cell pool and, frequently, established cardiomyopathy. This point is important when comparing younger ambulatory cohorts with older or non-ambulatory patients, and when asking why some therapies appear more effective in younger children.

Rather than cataloging platforms, the review is organized around linked questions. What does meaningful dystrophin restoration actually require? Why did AAV-microdystrophin become the first dominant platform despite being biologically incomplete? What did preclinical models establish, and what did they leave unaddressed? Why are clinical trials better understood as tests of an entire therapeutic system than of an isolated drug? Why has immune management become part of the therapeutic problem rather than a purely supportive measure? And what kind of convergence among gene transfer, splice modulation, vector engineering, and editing is plausible over the coming decade?

## 2 DMD as a distributed mechanical and regenerative disorder rather than a binary protein deficiency

The intuitive appeal of dystrophin restoration rests on a simple gene-to-disease relationship. The underlying biology is more layered. Dystrophin is not an inert structural filler; it links the intracellular actin cytoskeleton to the extracellular matrix through the dystrophin-associated glycoprotein complex (DGC), contributing to membrane stability, force transmission, and the localization of associated signaling proteins during repeated contraction (Liu S. et al., 2025; Wan et al., 2025). Its absence therefore initiates a cascade rather than a single lesion.

At the level of the individual fiber, early consequences include membrane fragility, abnormal calcium handling, reduced mechanical resilience, and mislocalization of signaling proteins such as neuronal nitric oxide synthase (nNOS) (Allen et al., 2016; Le et al., 2018). The process does not remain confined to injured fibers. Repeated cycles of damage and incomplete repair drive chronic inflammatory activation, extracellular matrix remodeling, fibro-fatty replacement, and eventual reorganization of muscle architecture (Broomfield et al., 2021; Florczyk-Soluch et al., 2021). Respiratory insufficiency and cardiomyopathy are best understood as components of the same progressive process rather than as unrelated late complications (Birnkrant et al., 2018; Meyers and Townsend, 2019).

These features have direct therapeutic implications. If DMD were a static protein deficiency, correction of a subset of fibers might be expected to produce a proportional clinical response. In practice, therapy is introduced into tissue already altered by years of degeneration and maladaptive repair. A corrected fibre may express dystrophin normally yet still transmit force through an endomysium expanded by collagen, operate within a chronically inflamed interstitium, be supplied by a capillary bed with reduced activity-dependent vasodilatation, and lie adjacent to uncorrected fibres that continue to degenerate and release damage-associated signals. This is one reason the relationship between molecular restoration and clinical outcome is unlikely to be linear.

Tissue distribution is a related and underweighted consideration. DMD affects all striated muscle, but different muscles contribute unequally to clinical milestones; loss of ambulation, decline in upper-limb function, respiratory failure, and cardiomyopathy follow distinct though overlapping trajectories. Consequently, restoration in a biopsy-accessible skeletal muscle cannot be interpreted in isolation. A therapy that performs well in the quadriceps but poorly in the diaphragm or heart may yield a molecular profile that overstates systemic benefit, whereas modest restoration across many muscle groups may matter more than marked restoration in one. The disease therefore requires a clear distinction between local pharmacodynamics and system-level efficacy.

The heart warrants particular attention. As supportive respiratory care has extended survival, DMD-associated cardiomyopathy has become an increasingly important determinant of outcome (Lechner et al., 2023; Gandhi et al., 2024). Experimental work has raised the possibility that improving skeletal muscle function without corresponding cardiac benefit could increase circulatory demand on a myocardium that remains dystrophin-deficient (Townsend et al., 2008; 2009; Gandhi et al., 2024). This concern does not invalidate skeletal-muscle-directed therapy, but it supports treating cardiac biodistribution as a primary efficacy consideration rather than a secondary one.

The central nervous system constitutes a further compartment, and one that the framing of DMD as a striated-muscle disease has tended to obscure. The DMD locus contains internal promoters generating a series of shorter isoforms with distinct spatial and temporal expression: full-length Dp427 in cortical and hippocampal neurons and cerebellar Purkinje cells, Dp260 in retina, Dp140 in developing brain and kidney, Dp116 in Schwann cells, and Dp71, the most abundant brain isoform, in glia and perivascular astrocytes (Byers et al., 1993; D’Souza et al., 1995; Lidov et al., 1995; Doorenweerd et al., 2017). Loss of these isoforms is not a marginal feature of the disease. Mean full-scale IQ in DMD is shifted downward by approximately one standard deviation, roughly a third of boys meet criteria for intellectual disability, and over 40% carry a diagnosis of autism spectrum disorder, attention-deficit hyperactivity disorder, obsessive-compulsive disorder, or a combination of these (Cotton et al., 2001; Hendriksen and Vles, 2008; Snow et al., 2013; Banihani et al., 2015; Ricotti et al., 2016). The burden follows a genotype gradient: mutations distal enough to abolish Dp140 in addition to Dp427 are associated with more severe cognitive and behavioral involvement, and approximately 90% of individuals lose Dp427 alone or Dp427 together with Dp140 (Pane et al., 2012; Chamova et al., 2013; Ricotti et al., 2016). Mechanistically, neuronal dystrophin anchors GABA_A receptor clusters at inhibitory synapses in hippocampus, amygdala and cerebellum, and its absence produces reduced receptor clustering, altered synaptic plasticity and exaggerated defensive responses in dystrophin-deficient mice (Knuesel et al., 1999; Sekiguchi et al., 2009; Waite et al., 2012; Vaillend and Chaussenot, 2017). The neurological phenotype therefore has the same molecular origin as the muscle phenotype but differs in character—it is largely developmental and non-progressive rather than degenerative. This implies both that any brain-directed intervention would have to precede the developmental window in which the deficit becomes established, and that a deficit already present in a school-age child may not be reversible even if dystrophin were restored in neurons.

The regenerative dimension adds further complexity. Satellite-cell dysfunction, altered division, mitochondrial abnormalities, impaired autophagy, and progressive loss of regenerative capacity have all been implicated in DMD (Chu et al., 2024; Franzmeier et al., 2025; Granet et al., 2025). This reframes the therapeutic goal: the aim is not only to replace a missing component in stable tissue but to intervene in tissue whose capacity for self-maintenance is declining. Durability therefore matters in two senses—the therapeutic event must persist, and the tissue must retain enough regenerative potential to benefit from it.

Understood in these terms, several limitations of current approaches become more interpretable. Counting dystrophin-positive fibers is not, in itself, an endpoint; younger and older patients may respond differently; one-time treatment is both attractive and biologically constrained; and vector performance, immune response, and disease stage cannot be evaluated independently. The disease arises at a single locus and converges on a single missing protein, but neither its mutation spectrum nor its clinical expression is uniform, and its treatment requirements are correspondingly multidimensional. A further element of tissue state is pharmacological: essentially every treated child has been on chronic glucocorticoids for years, which alters inflammatory tone, fibrosis, growth and immune competence, so the ‘tissue’ a vector enters is a steroid-modified one (Section 8.1).

## 3 What meaningful dystrophin restoration requires: quantity, structure, distribution, and persistence

A recurring source of confusion is the tendency to discuss “restoring dystrophin” as if it were a single measurable event. At minimum, four variables must be considered together: the amount of protein produced, its structural form, its spatial distribution, and the duration of expression. Many disagreements in the field can be traced to the conflation of these variables.

### 3.1 Amount is necessary but not self-interpreting

Observations in Becker muscular dystrophy first indicated that partial dystrophin can be clinically valuable: in-frame deletions permit synthesis of internally shortened proteins that retain partial function and are often associated with a substantially milder phenotype (Monaco et al., 1988; Aartsma-Rus et al., 2006; Deburgrave et al., 2007). The relevant lesson was not simply that some dystrophin is preferable to none, but that muscle can tolerate considerable internal truncation if critical domains are retained (Campuzano-Donoso et al., 2026).

The relationship between quantity and clinical effect is nonetheless not straightforward. Residual dystrophin above zero has been associated with milder disease (de Feraudy et al., 2021), and higher levels have been associated with greater skeletal protection in genotype–phenotype analyses (Neri et al., 2007). However, quantitative estimates depend on methodology: Western blot, immunofluorescence, fiber positivity, and localization studies measure different things, and none directly reports organ-level biomechanical benefit (Aartsma-Rus and Arechavala-Gomeza, 2018). A trial can therefore report apparently favorable molecular values while leaving functional significance uncertain. This does not render surrogate measures uninformative; it indicates that they must be interpreted within a broader biological framework. Because localization is reliably correct wherever expression occurs, the discriminating quantity is breadth: a modest level of protein achieved across many fibres and many muscles may be more meaningful than a higher level confined to a patchy subset of fibres or to a single sampled muscle, whereas an increase that is statistically measurable but below the functional threshold may be mechanistically genuine while producing no detectable change in timed-function tests or NSAA over one to 2 years.

### 3.2 The restored protein is not interchangeable

Dystrophin is a large modular protein, and not every truncated form is functionally equivalent (Elasbali et al., 2024). The N-terminal actin-binding domain, selected spectrin-like repeats, hinge regions, and the cysteine-rich domain that mediates interaction with β-dystroglycan are commonly retained in therapeutic designs because they support membrane anchoring and DGC assembly, whereas large portions of the rod domain and, in some contexts, the C-terminal domain can be removed with only partial functional loss (Bez Batti Angulski et al., 2023; Montagna et al., 2025). This does not imply that any construct within AAV packaging limits performs equally. Functional capacity depends on domain arrangement, repeat phasing, protein stability, and the ability to recruit binding partners, and constructs differ measurably in specific force, in resistance to eccentric contraction-induced injury, and in the extent to which they normalise centrally nucleated fibre counts and serum creatine kinase in mdx muscle (Banks et al., 2010; Ramos et al., 2019). For example, most clinically tested constructs omit spectrin repeats R16–R17 and therefore do not restore sarcolemmal nNOS anchoring; this predicts persistent loss of activity-dependent vasodilatation, with exercise-induced functional ischaemia and post-exertional fatigue—consequences that a supervised 10-m walk/run or a rested NSAA is unlikely to register (Li et al., 2010), and the possibility that larger ‘midi’ or full-length dystrophins better preserve diaphragmatic force generation and left-ventricular systolic function over multi-year follow-up merits direct head-to-head testing rather than dismissal on packaging grounds (Zhou et al., 2024; Tasfaout et al., 2025). The practical point is that deleting or repositioning a single hinge or spectrin repeat can substantially alter force transmission and DGC recruitment, because the therapeutic objective is a molecule that mechanically couples the cytoskeleton to the matrix, not immunodetectable protein at the sarcolemma. One clinical cohort does provide a comparator in which no domains are missing at all—patients with exon 2 duplications treated by U7-mediated splice correction, in whom the restored protein is full-length wild-type dystrophin (Section 7.2). It is currently the field’s only opportunity to ask empirically whether structural completeness translates into functional superiority.

### 3.3 Distribution is a question of coverage, not of correct localization

Distribution is a third variable, and it requires a distinction that is often collapsed in discussion of these therapies. Subcellular targeting is not the problem: therapeutically expressed dystrophin and microdystrophin localize to the sarcolemma in the expected pattern and recruit DGC components, and this has been reproducibly demonstrated for both antisense-mediated exon skipping and AAV gene transfer (Cirak et al., 2011; Mendell et al., 2013; 2020; Zaidman et al., 2023). Correct localization should therefore be regarded as an expected property of these platforms rather than as evidence of adequacy—and its converse holds as well: a construct can be correctly localized and still be functionally incomplete if it lacks domains such as R16–R17 (Section 3.2), so localization neither predicts nor substitutes for mechanical competence. What varies between platforms, doses and patients is instead the extent of coverage, and it varies on three nested scales: the fraction of myonuclei corrected within a single fibre, the fraction of fibres corrected within a muscle, and the fraction of the relevant tissue compartments—limb muscle, diaphragm, myocardium, and, by construct design, brain—reached at all. Dystrophin is organized through myonuclear domains and is not produced uniformly from a single nuclear source; its distribution along the sarcolemma has microanatomical structure, including enrichment at mechanically stressed sites such as the myotendinous junction (Morin et al., 2023), so the first of these scales is biologically meaningful rather than merely technical. This is what complicates interpretation of therapeutic expression. Antisense oligonucleotides can, in principle, induce exon skipping across many myonuclei, producing correctly localized but low-abundance restoration that is relatively evenly spread along the fibre, whereas vector-based approaches may transduce only a subset of nuclei when delivery is incomplete, yielding expression that is equally correctly localized where it occurs but mosaic in its coverage along the fibre (Morin et al., 2023). Whether mosaic rescue along a single fibre confers mechanical protection equivalent to uniform expression—and what proportion of myonuclei within a fibre must be corrected to stabilise it—remains unresolved. The relative preservation seen in many female carriers suggests that complete uniformity is not strictly required for functional protection, but carriers differ from boys with established disease in two respects that are difficult to reconcile: their mosaic expression is present from development onward rather than introduced into muscle that has already undergone years of degeneration, and they are oestrogen-exposed, which influences membrane repair and fibrotic remodelling (Vang et al., 2021).

Tissue-level distribution is at least as important as subcellular distribution. A therapy producing robust skeletal transduction but limited cardiac penetration may achieve partial functional benefit while leaving a major cause of morbidity largely unaddressed. The same reasoning extends to the central nervous system, with an additional constraint: the blood–brain barrier excludes most systemically administered oligonucleotides altogether, and the muscle-restricted promoters used in current clinical microdystrophin constructs are designed precisely to prevent expression outside striated muscle. Tissue-level distribution in DMD is therefore not simply a matter of how far a vector travels; for the brain it is also a matter of what the construct has been engineered to permit (Section 9.2). Because human cardiac tissue is rarely sampled, myocardial transduction itself is inferred rather than measured. This does not mean the heart is unobservable: cardiovascular magnetic resonance (CMR) with late gadolinium enhancement, native T1 and T2 mapping, extracellular volume (ECV) quantification and strain analysis, together with circulating high-sensitivity cardiac troponin I (hs-cTnI), provide validated, quantitative and serially repeatable readouts of myocardial fibrosis, edema and ongoing myocyte injury in DMD (Hor et al., 2009; Hor et al., 2013; Olivieri et al., 2016; Soslow et al., 2016; Voleti et al., 2020). What these modalities report, however, is downstream tissue state rather than transgene delivery, and they respond over months to years. They therefore constrain the plausible range of cardiac benefit without confirming that the vector reached the myocardium at a therapeutically relevant density, and the resulting uncertainty should not be mistaken for evidence of adequacy.

### 3.4 Persistence may become the dominant variable

Duration is the fourth consideration. AAV-mediated gene transfer offers one-time administration and sustained episomal expression in post-mitotic cells. However, dystrophic skeletal muscle is not reliably post-mitotic: fibers degenerate and regenerate, satellite cells are activated, and nuclei turn over (Le Hir et al., 2013). If an episomal cassette does not persist through this turnover, expression may decline as corrected fibers are replaced by newly formed, uncorrected tissue. The same concern applies to AAV-delivered exon-skipping cassettes such as U7-based systems, for which genome loss from dystrophic muscle has been demonstrated directly (Vulin et al., 2012; Le Hir et al., 2013), and for which expression may additionally decline through dilution by newly accreted myonuclei and through epigenetic silencing of the episomal genome (Roberts, 2023); persistence is, for that platform, the principal rather than an additional limitation (Section 7.2). Long-term durability therefore remains one of the most important open questions; AAV-based therapies may be clinically useful even if not permanent, but their value differs substantially depending on whether expression is maintained for 3–5 years or for the two or more decades over which the disease progresses, since a single infusion given at age 5 whose effect wanes by age 10 leaves the patient with no redosing option. Genome editing is attractive largely because it could, in principle, address this limitation at the level of the endogenous locus, particularly if satellite cells were reached, though this remains an aspiration rather than an established outcome in patients. This aspiration has recently begun to move toward direct clinical testing. An open-label, multidose dose-escalation study of an AAV-delivered CRISPR-mediated double-strand-break editing therapy in DMD patients has entered clinical evaluation, representing the first AAV-CRISPR gene-editing platform to reach human trials in this disease, with the first patient dosed in December 2024 (ClinicalTrials.gov identifier NCT06594094). Whether editing efficiency and durability observed in this early cohort will translate into a favorable risk–benefit profile remains to be established, but the transition from preclinical aspiration to first-in-human dosing marks a meaningful inflection point for this modality. At the same time, entry into the clinic has sharpened concern about genomic and transcriptomic consequences specific to double-strand-break-based editing delivered by AAV: because AAV genomes can integrate at double-strand-break sites, combining AAV delivery with nuclease-mediated cutting introduces an additional safety dimension that is largely absent from splice-modulation or episomal gene-transfer platforms (Jia et al., 2025).

In practical terms, meaningful dystrophin restoration is better defined as a four-dimensional state—adequate amount, adequate structure, appropriate tissue and subcellular distribution, and sufficient persistence—than as a single molecular event (Figure 1). Evaluations that reduce these dimensions to one readout risk misinterpreting both progress and failure.

## 4 Why AAV-microdystrophin emerged first: feasibility rather than optimality

Among the strategies developed for DMD, AAV-mediated microdystrophin gene transfer was the first to achieve substantial regulatory traction, largely because it fitted a functionally competent dystrophin surrogate within the ∼4.7-kb packaging capacity of a single intravenously administrable AAV vector, accepting a truncated protein in exchange for body-wide delivery. It advanced not because it resolved every relevant problem, but because the alternatives were less mature: dual-vector reconstitution of larger dystrophins was inefficient and stoichiometrically unreliable, *in vivo* editing lacked a validated delivery route and carried unresolved off-target and integration liabilities, and cell-based approaches had not achieved engraftment across a tissue compartment of this size. The specific design, dosing and safety characteristics of the constructs that reached patients are set out in Tables 1, 2 and discussed in Section 4.2 and Section 4.3.

**TABLE 1:** Micro- and mini-dystrophin constructs evaluated in patients: cassette design, dose, and development status.

Product (INN/code)	Sponsor	Capsid	Promoter/regulatory elements	Transgene: retained domains	nNOS repeats (R16–R17)	CT domain	Route and dose (vg/kg)	Key trials (registry ID)	Status
rAAV2.5-CMV-minidystrophin	Nationwide Children’s/Asklepios	rAAV2.5 (engineered AAV2 with AAV1 surface residues)	CMV (ubiquitous)	Mini-dystrophin (ΔR4–R23/ΔCT-type)	No	No	Intramuscular (biceps), escalating (∼10^10^ vg per muscle)	Phase 1 (NCT00428362)	Completed; transient, local expression; transgene-directed T-cell response (Mendell et al., 2010)
Delandistrogene moxeparvovec (SRP-9001; ELEVIDYS)	Sarepta/Roche	rAAVrh74	MHCK7 (α-MHC enhancer + muscle CK promoter)	ABD1, H1, R1–R3, H3, R24, CR, H4	No	No	IV, 1.33 × 10^14^ (linear-genome titration; ≈2 × 10^14^ by earlier supercoiled qPCR)	Study 101 (NCT03375164); Study 102 (NCT03769116); ENDEAVOR/103 (NCT04626674); EMBARK/301 (NCT05096221); ENVISION/303 (NCT05881408)	FDA accelerated approval 2023; expanded 2024; boxed warning and indication narrowed to ambulatory ≥4 years, November 2025
Fordadistrogene movaparvovec (PF-06939926)	Pfizer	AAV9	Human muscle-specific (creatine-kinase-based) promoter	Mini-dystrophin (truncated; nNOS repeats not retained)	No	No	IV, 1 × 10^14^ and 2 × 10^14^ (phase 3 at 2 × 10^14^)	Phase 1b (NCT03362502); CIFFREO phase 3 (NCT04281485)	Discontinued 2024 after CIFFREO missed primary and secondary endpoints; serious safety events (see Table 2)
SGT-001	Solid Biosciences	AAV9	CK8/CK8e (synthetic muscle-restricted)	Microdystrophin retaining the nNOS-binding region	Yes	No	IV, 5 × 10^13^ and 2 × 10^14^	IGNITE DMD phase I/II (NCT03368742)	Deprioritized after complement-mediated SAEs and clinical holds; variable expression
SGT-003	Solid Biosciences	AAV-SLB101 (rationally engineered myotropic capsid)	CK8e	Microdystrophin retaining the nNOS-binding region	Yes	No	IV, 1 × 10^14^	INSPIRE DUCHENNE phase I/II (NCT06138639)	Ongoing; early interim expression and CK data reported
RGX-202	Regenxbio	AAV8	Spc5-12 (synthetic muscle-specific)	Microdystrophin including a C-terminal domain segment	No	Partial	IV, 1 × 10^14^ and 2 × 10^14^	AFFINITY DUCHENNE phase I/II/III (NCT05693142)	Ongoing pivotal expansion; prophylactic immunosuppression per protocol
GNT0004	Genethon	AAV8	Spc5-12	Microdystrophin (MD1; ΔR4–R23/ΔCT-type)	No	No	IV, ∼1 × 10^13^ and ∼3 × 10^13^ (an order of magnitude below other systemic programmes)	First-in-human phase I/II/III	Ongoing; functional stabilization reported in a small effective-dose cohort

ABD1, N-terminal actin-binding domain; CK, creatine kinase; CR, cysteine-rich domain; CT, C-terminal domain; H, hinge; MHC, myosin heavy chain; R, spectrin-like repeat. All cassettes are flanked by AAV2 inverted terminal repeats. Doses are as reported by sponsors and are not strictly comparable across programmes because vector-genome titration is assay-dependent (Section 9.5).

**TABLE 2:** Toxicities and serious adverse events reported in clinical micro-/mini-dystrophin programmes, grouped by mechanism.

Programme	Hepatic	Complement/TMA	Transgene-directed muscle immunity	Cardiac	Deaths	Regulatory/programme consequence
rAAV2.5-CMV-minidystrophin (IM)	Not applicable at IM doses	Not reported	Dystrophin-specific T-cell responses detected, including in one patient before dosing (revertant-fibre priming)	Not applicable	None	Established transgene antigenicity as a design constraint (Mendell et al., 2010)
Delandistrogene moxeparvovec	Transaminase elevation common; acute serious liver injury; fatal acute liver failure in non-ambulatory patients	Reported infrequently	Immune-mediated myositis, including severe cases with bulbar/respiratory weakness; associated with deletions in the exon 8/9 region	Troponin elevation; myocarditis reported	≥2 from acute liver failure (non-ambulatory); shipments suspended mid-2025	Boxed warning (November 2025); indication narrowed to ambulatory ≥4 years; exon 8 and/or 9 deletions excluded; weekly LFTs ≥3 months
Fordadistrogene movaparvovec	Transaminase elevation	Complement activation and thrombotic microangiopathy, with thrombocytopenia, haemolysis and acute kidney injury	Immune-mediated myositis reported	Fatal cardiogenic event in a non-ambulatory participant (crossover cohort)	≥2 (one peri-infusion cardiac; one later in follow-up)	Trial pause and protocol amendment; programme terminated after CIFFREO failure
SGT-001	Transaminase elevation	Complement activation/TMA, grade 4 events (thrombocytopenia, haemolysis, AKI, cardiopulmonary insufficiency)	Not the dominant liability	Cardiopulmonary insufficiency in the context of TMA	None reported	Repeated FDA clinical holds; programme deprioritized in favour of SGT-003
SGT-003	Transaminase elevation managed with immunosuppression	No complement-mediated SAEs reported in early cohorts to date	None reported to date	None reported to date	None reported	Ongoing
RGX-202	Transaminase elevation	No TMA reported to date	None reported to date	None reported to date	None reported	Ongoing
GNT0004	Transaminase elevation	Complete safety dataset not yet peer-reviewed	Not yet reported	Not yet reported	None reported	Ongoing at substantially lower doses

AKI, acute kidney injury; IM, intramuscular; LFT, liver function test; SAE, serious adverse event; TMA, thrombotic microangiopathy. Entries reflect publicly available peer-reviewed reports, regulatory labelling and sponsor communications; the depth of disclosure differs markedly between programmes, and absence of a reported event in an ongoing trial should not be read as evidence of absence.

The central constraint is well established. Full-length dystrophin exceeds conventional AAV packaging capacity: the coding sequence is approximately 11 kb and the gene spans about 2.4 Mb across 79 exons, whereas recombinant AAV accommodates roughly 4.7 kb including regulatory elements (Koenig et al., 1988; Chamberlain, 2002). This mismatch is a defining design pressure: any AAV-based approach must reduce the cargo, split it across vectors, or use a different delivery system.

The earliest broadly workable solution was to reduce the cargo. Genotype–phenotype data from Becker muscular dystrophy and transgenic studies indicated that dystrophin retains meaningful function after substantial internal deletion, provided key structural elements are preserved (England et al., 1990; Phelps et al., 1995). This informed the design of mini- and microdystrophins engineered to fit within AAV limits while retaining domains considered most important for membrane stabilization and DGC interaction. Microdystrophin was thus not proposed as an improvement over full-length dystrophin, but as the largest biologically credible protein deliverable in a single AAV vector.

AAV offered additional practical advantages: relatively low pathogenicity, sustained expression in muscle, and, for certain serotypes, useful tropism toward striated muscle (Zincarelli et al., 2008). Muscle-restricted promoters such as MHCK7, CK8/CK8e, and Spc5-12 improved the platform further by limiting ectopic expression and the associated risk of immune activation (Li et al., 1999; Salva et al., 2007; Wang et al., 2008). This reflected a broader recognition that, in DMD, vector pharmacology and immunology are closely linked.

The platform’s limitations should be stated equally clearly. AAV-microdystrophin advanced by circumventing the full-length cargo problem, not by removing the other barriers. Because the target tissue mass is large, body-wide delivery requires high systemic doses, which place substantial demands on the liver and immune system (Duan, 2018; Chamberlain et al., 2025). Anti-capsid immunity generally precludes straightforward redosing, so treatment is effectively single-use (Goedeker et al., 2023; Verma et al., 2023). The transgene remains episomal, leaving durability uncertain in regenerating muscle (Vulin et al., 2012; Bengtsson et al., 2021). And because microdystrophin is structurally incomplete, some native functions of full-length dystrophin may be only partially restored even when transduction succeeds (Li et al., 2010; Ramos et al., 2019). Relative to the alternatives available two decades ago, the approach was well engineered; relative to the biological demands of DMD, it was expected to be incomplete. Much of the disagreement about the clinical record follows from retaining one of these statements and discarding the other—reading demonstrated expression as proof of adequacy, or a missed functional endpoint as proof that the mechanism is invalid.

A further reason for early adoption was alignment with existing manufacturing and regulatory infrastructure. AAV production, purification, and potency assessment were maturing in other indications; DMD-specific challenges—full-versus-empty capsid ratios, batch consistency, and the very large quantities required for systemic infusion—remained substantial but were, at least, addressable within an existing manufacturing framework (El Andari et al., 2022). By contrast, more biologically complete approaches such as full-length reconstruction or *in vivo* editing depended on less mature delivery systems or multicomponent designs with slower development paths. AAV-microdystrophin is therefore best interpreted as a transitional advance of considerable importance: it demonstrated that body-wide molecular intervention is achievable in patients, and it also made clear that feasibility is not equivalent to sufficiency.

### 4.1 How the systemic dose was arrived at: an exposure range delimited by spinal muscular atrophy, refined by DMD-specific dose-escalation, but never approached from below in patients

A further reason that systemic AAV-microdystrophin advanced when it did, and one that is rarely stated explicitly, is that a dosing framework was already available. The first systemic AAV programme to demonstrate efficacy in a neuromuscular disease was AAV9-mediated SMN1 replacement in spinal muscular atrophy (SMA) type 1, in which intravenous self-complementary AAV9 at 6.7 × 10^13^ and 2.0 × 10^14^ vg/kg produced survival free of permanent ventilation and acquisition of motor milestones in infants (Mendell et al., 2017), leading to registration and to confirmatory phase 3 trials conducted at 1.1 × 10^14^ vg/kg (Day et al., 2021; Mercuri et al., 2021). Two elements of that experience were transferred more or less intact into DMD. The first was an empirical range for systemic vector exposure: nonclinical work within the same programme, including dose-limiting hepatic and dorsal root ganglion toxicity in nonhuman primates and piglets at 2 × 10^14^ vg/kg (Hinderer et al., 2018), together with the subsequent finding at higher doses in X-linked myotubular myopathy that 3 × 10^14^ vg/kg produced fatal hepatobiliary events (Wilson and Flotte, 2020; Shieh et al., 2023), positioned approximately 1–2 × 10^14^ vg/kg as simultaneously the lowest systemic dose with demonstrated clinical efficacy and the approximate upper limit of tolerability. The second was a peri-infusion management template: prophylactic corticosteroids beginning shortly before infusion together with serial transaminase surveillance, adopted after transaminase elevations and, subsequently, cases of acute liver injury and fatal acute liver failure were reported in treated infants (Feldman et al., 2020; Chand et al., 2021). Systemic DMD programmes entered the clinic inside this framework: the doses selected clustered in the 1 × 10^14^–2 × 10^14^ vg/kg range, and the steroid prophylaxis and hepatic monitoring schedules were closely modelled on SMA practice.

It would be inaccurate, however, to describe the dose used in DMD as having been adopted without disease-specific justification, and the sequence by which it was arrived at is worth setting out explicitly. Within the exposure envelope that SMA had delimited, formal dose-escalation and toxicity assessment were performed in the mdx mouse for the construct that reached patients first: intravenous rAAVrh74.MHCK7.micro-dystrophin was administered at low (2 × 10^12^ vg total; ≈8 × 10^13^ vg/kg), intermediate (6 × 10^12^vg; ≈2 × 10^14^ vg/kg) and high (1.2 × 10^13^ vg; ≈6 × 10^14^ vg/kg) doses, and the intermediate dose was the lowest at which specific force in the diaphragm and tibialis anterior reached wild-type values, accompanied by reduced fibrosis and central nucleation and normalisation of myofibre size, with no clear additional functional gain at the high dose (Potter et al., 2021). That result, rather than the SMA precedent alone, defined the dose carried into the first systemic DMD trial, in which four boys received 2.0 × 10^14^ vg/kg with corticosteroid prophylaxis and showed robust microdystrophin expression, reduced creatine kinase and functional stabilisation without dose-limiting toxicity (Mendell et al., 2020). Later programmes followed the same logic: RGX-202 entered the clinic at the minimum effective dose of 1 × 10^14^ vg/kg identified in mdx dose-ranging studies spanning 3× 10^13^–5 × 10^14^ vg/kg (Owusu et al., 2026). Independent convergence on the same order of magnitude has also come from a mechanistically unrelated AAV strategy in the same disease: surrogate gene therapy with rAAVrh74.MCK.GALGT2, delivered by intravascular limb infusion at 2.5 × 10^13^ and 5 × 10^13^ vg/kg per leg (5 × 10^13^ and 1 × 10^14^ vg/kg in total), was tolerated without serious adverse events, indicating that the 5× 10^13^–2 × 10^14^ vg/kg region is not an artefact of one construct, one sponsor, or the SMA precedent (Flanigan et al., 2022). The dose used in DMD is therefore best described as an exposure envelope whose upper limit was constrained by toxicity observed in other indications, and whose effective range was subsequently established within DMD itself—in mdx mice, in the first systemic DMD trial, and by an independent AAV strategy in the same disease—rather than as a value simply transposed from SMA.

What was inherited, and what was not, should nonetheless be distinguished carefully. The nonclinical dose–response work described above was conducted inside the exposure envelope that SMA had already delimited, and the peri-infusion corticosteroid and transaminase-monitoring template was adopted essentially unchanged; neither the upper bound nor the immune-management schedule was re-derived from first principles in DMD. Two assumptions travelled with that envelope which DMD does not satisfy. The first is that vg/kg is a transferable unit. SMA was dosed in infants weighing roughly 3–8.5 kg, whereas systemically treated boys with DMD typically weigh 15–40 kg or more, so a nominally identical vg/kg dose corresponds to a five-to tenfold greater absolute load of vector genomes and capsid protein per patient, with proportionally greater demands on hepatic processing, innate immune tolerance, and manufacturing capacity. The second concerns the target. In SMA, transduction of a numerically small population of motor neurons is sufficient, and the target tissue is a negligible fraction of body mass; in DMD the target is striated muscle, approximately a third of body mass, so the same vg/kg dose must be distributed across an incomparably larger compartment. Equivalence expressed in vg/kg therefore implies neither equivalence in the biological demand placed on the dose nor equivalence in the immunological burden it imposes.

Three consequences follow, and they bear directly on how the safety record should be read. First, although a minimum effective dose was defined for DMD, it was defined in the mouse: the ascending series was conducted in a small, comparatively mildly affected animal, and the dose taken into patients was the murine minimum effective dose rather than the lowest dose shown to be effective in a child. Clinically, the range has therefore been approached from above rather than from below; with the partial exception of GNT0004, which explored substantially lower doses and reported functional stabilisation in a small effective-dose cohort, systemic programmes began at or near the top of the envelope, and no clinical dose–response study has established the exposure at which benefit is first lost. It follows that the hepatic, complement-mediated and transgene-directed toxicities discussed in Section 8 have been observed at doses not shown in patients to be the minimum required for benefit, so the therapeutic window in this indication remains poorly defined at its lower edge. Finally, because the SMA precedent was established in immunologically naive neonates, whereas DMD is treated in older children who are chronically corticosteroid-exposed, more frequently anti-AAV seropositive, and already carrying myocardial and hepatic consequences of the disease itself, the tolerability margin observed in SMA had no strong reason to transfer intact—an expectation that the subsequent DMD safety record has borne out.

A concrete illustration that the inherited ceiling is not a physical requirement is provided by the AAV-U7 exon 2 skipping programme, whose minimally efficacious systemic dose in the Dup2 mouse was 3 × 10^13^ vg/kg—approximately an order of magnitude below the range used across microdystrophin programmes ((Roberts, 2023) Section 7.2). Cargo size, expression requirements and self-complementary genome configuration all differ, so this is not a transferable dose; it does, however, demonstrate that therapeutically relevant systemic AAV exposure in DMD exists well below the boundary imported from SMA.

### 4.2 Construct and cassette design across clinical programmes: what actually differs, and what it predicts

The programmes summarised in Table 1 are frequently discussed as a single therapeutic class, but they differ along at least four design axes, and each difference generates a testable functional prediction. Setting them out explicitly is useful because it converts an apparently uniform “microdystrophin platform” into a set of partially independent engineering choices whose consequences can, in principle, be distinguished clinically.

Promoter and regulatory context. The first clinical construct used a cytomegalovirus promoter, which is ubiquitously active and therefore drives transgene expression in antigen-presenting and non-muscle cells; this is one plausible contributor to the transgene-directed T-cell response observed in that trial (Mendell et al., 2010). Every subsequent systemic programme has used a muscle-restricted regulatory cassette—MHCK7, which couples an α-myosin heavy chain enhancer to a truncated muscle creatine kinase promoter and confers strong activity in skeletal muscle and heart (Salva et al., 2007); the synthetic CK8/CK8e series, which was optimised for compactness and muscle specificity (Wang et al., 2008); or Spc5-12, a synthetic promoter assembled from muscle-specific regulatory motifs with activity exceeding that of naturally occurring sequences (Li et al., 1999). The trade-off is explicit and, in our view, insufficiently acknowledged: restricting expression to striated muscle reduces ectopic antigen presentation and hepatic transgene expression, but it also excludes the central nervous system by design rather than by delivery failure (Section 9.2), and it makes the relative cardiac activity of MHCK7- versus Spc5-12- or CK8e-driven cassettes a clinically relevant, and currently untested, comparison.

Domain content. All clinical single-vector constructs retain the N-terminal actin-binding domain, hinge 1, a subset of proximal spectrin repeats, hinge 3 or 4, repeat R24 and the cysteine-rich domain, because these support sarcolemmal anchoring and β-dystroglycan binding (Section 3.2). They differ in two respects that are functionally, not cosmetically, significant. The first is whether spectrin repeats R16–R17 are retained: these are required for sarcolemmal nNOS anchoring, and constructs lacking them are not expected to restore activity-dependent vasodilatation (Li et al., 2010). SGT-001 and SGT-003 retain this region; delandistrogene moxeparvovec, fordadistrogene movaparvovec and GNT0004 do not. The second is whether any part of the C-terminal syntrophin/dystrobrevin-binding region is retained; RGX-202 includes a C-terminal segment intended to improve recruitment of associated proteins and resistance to contraction-induced injury (Owusu et al., 2026), whereas the other clinical constructs omit it. Hinge composition also differs between constructs and measurably alters force transmission in preclinical models (Ramos et al., 2019; Banks et al., 2020; Wasala et al., 2023). Because none of these constructs has been compared head to head in patients, and because the endpoints used in pivotal trials are rested, supervised functional tests unlikely to interrogate exercise-dependent perfusion, the field currently has no clinical evidence that discriminates between them.

Capsid. Three natural serotypes are represented (AAVrh74, AAV9, AAV8), together with one engineered myotropic capsid (AAV-SLB101 in SGT-003) and one early chimeric capsid (rAAV2.5). Serotype choice affects muscle and cardiac tropism, hepatic uptake and cross-reactivity with pre-existing antibodies (Zincarelli et al., 2008; Shen et al., 2022), and it is therefore not separable from either the dose required or the immunological burden incurred (Section 8 and Section 10.1).

Dose and immunosuppression. With the exception of GNT0004, which tested approximately 10^13^ vg/kg, all systemic programmes clustered at 1–2 × 10^14^ vg/kg—the range inherited from spinal muscular atrophy (Section 4.1) — and this clustering is the single most important reason that cross-programme differences in toxicity cannot be attributed cleanly to capsid or cassette. Peri-infusion immunosuppression also differs: regimens have ranged from short prophylactic corticosteroid courses modelled on SMA practice to extended multi-agent protocols incorporating additional immunosuppression, and these differences are rarely held constant when safety records are compared. Finally, cassette composition is disclosed inconsistently across products—intron, polyadenylation signal, codon-optimisation status and total cassette length are not uniformly reported—which limits independent interpretation of why nominally similar constructs behave differently. We regard standardised reporting of cassette architecture, titration method and immunosuppression schedule as a minimum requirement for meaningful cross-programme comparison.

### 4.3 The clinical safety record, read by mechanism rather than by sponsor

Table 2 summarises reported toxicities grouped by mechanism rather than by product, because the pattern that emerges is mechanistic rather than idiosyncratic. Four liabilities recur.

Hepatic injury is the most common clinically significant event and is not uniformly manageable. Transaminase elevation occurs across all systemic programmes and usually responds to corticosteroids, but acute serious liver injury and fatal acute liver failure have occurred with delandistrogene moxeparvovec, predominantly in non-ambulatory patients, and this prompted the November 2025 boxed warning, the restriction of the indication to ambulatory patients aged 4 years and older, and mandatory weekly liver function monitoring for at least 3 months ((Taylor, 2025) Section 8.1).

Complement activation with thrombotic microangiopathy defines a second axis and was most prominent in the fordadistrogene movaparvovec and SGT-001 programmes, where thrombocytopenia, haemolysis and acute kidney injury followed a reproducible sequence rather than occurring randomly (Costa-Verdera et al., 2023; Byrne et al., 2025).

Transgene-directed immune-mediated myositis constitutes a third axis and is genotype-determined: patients whose deletions remove the exons encoding epitopes present in the therapeutic construct are not immunologically tolerized to those epitopes, which is why deletions in the exon 8/9 region are now an explicit contraindication in approved labelling ((Bönnemann et al., 2023; Potter et al., 2025) Section 8.2).

Cardiac events form a fourth axis and are the least well captured. A fatal cardiogenic event in a non-ambulatory participant, and the systematic troponin surveillance subsequently implemented, indicate that peri-infusion myocardial injury can occur and is detectable if sought (Sherlock et al., 2025), which is one reason we argue that cardiac biomarker and imaging surveillance should be treated as a safety requirement of systemic AAV administration in DMD and not solely as an efficacy measure (Section 9.1).

Two interpretive cautions apply to Table 2. First, the depth of public disclosure differs greatly between programmes: terminated and approved products have been reported in detail, whereas ongoing trials are represented largely by interim sponsor communications, so the apparently cleaner safety profiles of newer programmes partly reflect shorter follow-up and less complete reporting rather than demonstrably better tolerability. Second, because dose, capsid, cassette and immunosuppression regimen all differ simultaneously between programmes, and because dose itself was imported rather than derived (Section 4.1), the safety record cannot at present be resolved into product-specific attributions. This is precisely why we regard the ratio of corrected muscle mass to total immunological burden, rather than any single peak expression value, as the more informative measure of progress (Section 8.3).

## 5 What animal models established, and what they did not

Preclinical models were essential to the development of DMD gene therapy (Duan et al., 2021). They demonstrated that truncated dystrophins can restore membrane localization, recruit DGC components, reduce creatine kinase leakage, improve histology, and enhance muscle performance (Duan et al., 2021; Bez Batti Angulski et al., 2023). Systemic administration in *mdx* mice and, subsequently, in larger models such as the golden retriever muscular dystrophy (GRMD) dog moved the field from proof of principle to a credible translational program and provided the basis for human dosing (Le Guiner et al., 2017; Liu M. et al., 2025).

The same models also encouraged an overly linear view of therapy. In animal studies, the sequence from vector delivery to microdystrophin expression, membrane stabilization, and functional improvement can appear direct. The human experience has been less so—not primarily because the underlying biology is incorrect, but because of factors the models capture incompletely, such as host immunogenicity, persistent tissue fibrosis (Palmieri et al., 2026), and the functional limitations of truncated proteins in larger hearts (Hart et al., 2024).

The first is phenotype severity and timescale. The *mdx* mouse, though valuable, is comparatively mild: lifespan is near normal, fibrosis is limited in many muscles, and the cumulative cardiopulmonary burden does not match that of boys or young men with advanced disease (Le Guiner et al., 2017). Vector-mediated rescue in mice is therefore often observed against a more permissive tissue background than that of typical trial participants, so marked preclinical improvement does not guarantee a proportional human effect. The GRMD dog resembles human disease more closely and provided important evidence that systemic AAV-microdystrophin can improve histology, function, and respiratory parameters in a large animal (Le Guiner et al., 2017; Birch et al., 2023). Even so, experimental conditions remain more controlled than clinical practice, with defined treatment timing, constrained genetic background, and fewer comorbid variables, whereas patients differ in mutation class, steroid history, baseline function, prior immune exposure, fibrosis burden, and cardiac status.

The second is biodistribution. Systemic transduction in animals can be broad, but scaling from mouse to child is not straightforward. Vascular architecture, body size, immune exposure, and target tissue mass all influence the effective pharmacology of AAV, so a dose producing extensive correction in an animal may yield lower transduction density in a patient even when normalized to vector genomes per kilogram. This helps explain why expression in clinical biopsies has often been more mosaic than parts of the preclinical literature might suggest. This asymmetry is most acute for the heart. In animal models, cardiac transduction and microdystrophin expression can be quantified directly at necropsy, whereas in patients they must be inferred from imaging and circulating biomarkers that report myocardial tissue state rather than vector delivery (Section 9.1). Preclinical programs therefore generate a class of cardiac evidence that human studies structurally cannot reproduce, and confidence in cardiac efficacy has, to a considerable extent, been carried across this gap by extrapolation.

The third is immunity. Most preclinical systems do not reproduce the range of human anti-AAV serostatus (Goedeker et al., 2023; Verma et al., 2023), nor do they readily model the possibility that some *DMD* genotypes recognize microdystrophin sequences as foreign (Bönnemann et al., 2023; Potter et al., 2025). The observation that certain deletions predispose to transgene-directed immune reactions emerged from human trials rather than preclinical prediction and altered patient selection across programs (Bönnemann et al., 2023).

Finally, animal studies rarely confront the clinical endpoint problem. In mice and dogs, histology, protein expression, creatine kinase, and force measurements are sensitive and interpretable, whereas regulatory decisions in humans depend on timed functional tests, North Star Ambulatory Assessment (NSAA) change, respiratory trajectories, upper-limb measures, or external-control comparisons, each of which carries developmental variability and disease-stage confounding (Mendell et al., 2023; Mendell et al., 2025; Zaidman et al., 2023). Biological effects may therefore be real without immediately translating into a statistically unambiguous clinical endpoint.

These points do not diminish the value of preclinical data, which were sufficient to justify human development and continue to support innovation. They indicate that preclinical models answer a necessary but limited question—whether an intervention can work under favorable conditions—while human trials test a harder one: whether it still works when delivery, patient heterogeneity, immunity, and endpoint sensitivity all become limiting at once. Much of the current effort can be understood as an attempt to narrow the gap between these questions. Notably, this gap includes the biomarkers themselves: because cardiac and diaphragmatic transduction can be quantified directly at necropsy, animal studies are the only setting in which the non-invasive surrogates that later carry all human cardiac claims—hs-cTnI, native T1, ECV and strain—can be calibrated against measured delivery rather than assumed to reflect it.

## 6 Clinical development as a test of the whole therapeutic system

The clinical history of dystrophin-restoring therapy is often described as a series of company-specific successes and failures. That framing is understandable but incomplete. Taken together, trials over the past two decades have functioned as a distributed test of whether an entire therapeutic system—vector, promoter, transgene, dose, patient selection, immune management, endpoint design, and disease stage—can be aligned such that a molecularly active therapy produces a functional difference large enough, and early enough, to be detected by the endpoint against which it is being judged. Several apparent contradictions become more interpretable from this perspective (Figure 3).

**FIGURE 3:**
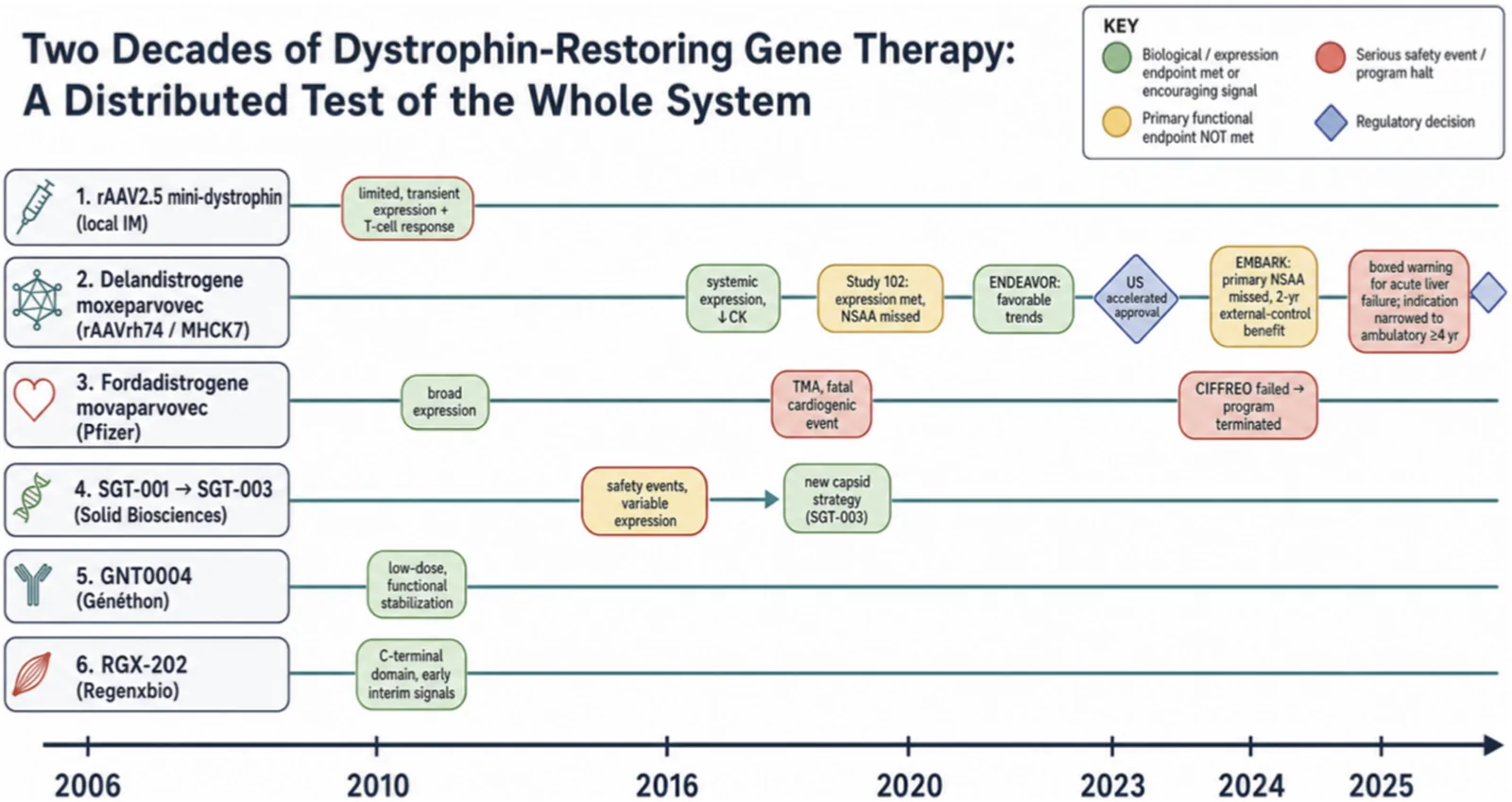
Clinical development of dystrophin-restoring gene therapy as a test of an entire therapeutic system, 2006–2025. Rather than a series of isolated company successes and failures, the trial record is better read as a distributed test of whether vector, promoter, transgene, dose, patient selection, immune management, endpoint design, and disease stage can be made coherent. For context, the exposure envelope within which these programmes operate (∼1–2 × 10^14^ vg/kg) was first delimited by the preceding intravenous AAV9 programme in spinal muscular atrophy, which also supplied the corticosteroid prophylaxis and transaminase monitoring template subsequently adopted in DMD; the specific doses taken into patients were then defined by dose-escalation in mdx mice and confirmed in early-phase DMD trials (Section 4.1). Where feasible, plotting the SMA phase 1 (2017) and phase 3 (2021) milestones as a preceding context band makes this dependency explicit. Markers are color-coded by outcome: biological/expression signals (green), missed primary functional endpoints (amber), serious safety events or program termination (red), and regulatory decisions (blue). Recurrent dissociation between reproducible molecular expression and inconsistent short-term functional endpoints, together with dose- and genotype-linked immune toxicities, culminated in the November 2025 boxed warning and narrowed indication for delandistrogene moxeparvovec—concretely illustrating that safety, disease stage, and efficacy cannot be evaluated independently. Figure 3 is intended as a chronological overview of how the field’s questions and constraints evolved, not as a repository of product-level detail; construct architecture, promoters, capsids, trial identifiers, doses and reported toxicities are tabulated in Tables 1, 2, to which this figure should be read as a companion. IM, intramuscular; CK, creatine kinase; NSAA, North Star Ambulatory Assessment; TMA, thrombotic microangiopathy; SMA, spinal muscular atrophy; XLMTM, X-linked myotubular myopathy; vg/kg, vector genomes per kilogram.

The earliest AAV-based attempt in patients was deliberately conservative. Local intramuscular administration of an rAAV2.5–CMV–mini-dystrophin construct (2006–2009) produced limited and transient expression (Mendell et al., 2010). In a narrow sense this was disappointing, but it was informative: it showed that delivering a dystrophin cassette into muscle is insufficient when expression is weak, distribution is local, and a T-cell response develops against the transgene product—an early indication that antigenic context, not delivery alone, would constrain the field (Mendell et al., 2010).

The first substantial systemic advance came with delandistrogene moxeparvovec, which uses an rAAVrh74 capsid and the MHCK7 promoter to drive microdystrophin expression (Mendell et al., 2020). In an early-phase setting it achieved body-wide intravenous administration with microdystrophin detectable in post-treatment biopsies, reduced serum creatine kinase, and NSAA trajectories numerically more favourable than matched natural-history data, in a small uncontrolled cohort (Mendell et al., 2020), establishing that clinically relevant systemic delivery was achievable in patients. As development expanded, the picture became more complex. Study 102 met its biological endpoint of increased microdystrophin expression but did not meet the primary clinical endpoint of NSAA change at week 48 in the intention-to-treat population (Mendell et al., 2023). ENDEAVOR, analysed against external natural-history comparators, reported microdystrophin expression by immunofluorescence and Western blot together with NSAA and timed-function values that declined more slowly than in the comparator cohort (Zaidman et al., 2023). The phase 3 EMBARK program again did not meet its primary week-52 NSAA endpoint, although several secondary measures numerically favored treatment and longer-term analyses against pre-specified external controls suggested benefit at 2 years (Mendell et al., 2025; Pascual-Morena et al., 2025). These results supported two competing interpretations: that the therapy was biologically active but had not yet demonstrated sufficient clinical effect, and that reliance on surrogate markers and subgroup analyses risked overstating efficacy. Both interpretations capture part of the evidence.

A measured reading is that delandistrogene moxeparvovec produced a genuine but variable effect within an imperfect trial framework. It reproducibly generated microdystrophin and plausibly slowed decline in at least some subgroups, but the magnitude, timing, and distribution of effect were not consistently large enough to yield a clear short-term result on every conventional endpoint in every cohort. The subsequent U.S. regulatory decisions in 2023 and 2024, including broadened access, reflected the tension between mechanistic plausibility and endpoint ambiguity (Hoy, 2023; Bhattacharyya et al., 2024). The controversy indicates not that the data were uninformative, but that the effect size lay in the range in which a genuine biological action cannot be reliably separated from measurement variability by a single 52-week functional endpoint in a cohort of this size.

Pfizer’s fordadistrogene movaparvovec program illustrated a related point from a different angle. Early trials showed broad expression and biochemical effects (Butterfield et al., 2025), suggesting that the microdystrophin concept was not platform-specific. However, serious safety events—including a fatal cardiogenic event in a non-ambulatory participant, cases of thrombotic microangiopathy, and a subsequent death during follow-up—altered the program’s trajectory (Philippidis, 2024; Byrne et al., 2025; Sherlock et al., 2025). When the phase 3 CIFFREO study did not meet its primary and secondary endpoints, development was terminated (Philippidis, 2024). These outcomes do not establish that microdystrophin cannot work; they indicate that vector dose, serotype, immune activation, and patient vulnerability can narrow the therapeutic window to the point that a mechanistically valid approach becomes clinically untenable.

Other programs add further nuance. Solid Biosciences moved from the earlier SGT-001 experience, which was affected by safety events and variable expression (Roberts et al., 2023), toward SGT-003 and newer capsid strategies intended to improve muscle targeting and reduce toxicity. Généthon’s GNT0004 programme emphasised markedly lower dosing—approximately an order of magnitude below the other systemic programmes—and reported encouraging functional stabilisation in a small effective-dose cohort (Table 1). Regenxbio’s RGX-202 incorporated a C-terminal domain segment intended to improve recruitment of associated proteins and resistance to contraction-induced damage (Owusu et al., 2026), with early interim signals. None of these should be interpreted as established solutions, but collectively they indicate that the field is exploring a multidimensional design space rather than evaluating a single, fixed drug class.

Read together, the trials support several conclusions. Systemic microdystrophin expression in human muscle is now reproducibly demonstrated. Functional efficacy, however, is conditional and depends on vector distribution, disease stage, transgene design, and—plausibly—cardiac coverage. Toxicity is not random; it tracks identifiable liabilities such as high vector dose, innate immune activation, complement involvement, and genotype-dependent transgene immunogenicity. One caveat applies throughout this section and the next. Doses are conventionally reported as vector genomes per kilogram (vg/kg), but this unit is not standardized across sponsors: titers determined by quantitative PCR are sensitive to amplicon position, template linearisation and self-complementary genome structure, and can differ from droplet digital PCR values by up to twofold or more for the same preparation (Fagone et al., 2012; Werling et al., 2015; Furuta-Hanawa et al., 2019). Reference materials exist but are inconsistently applied (Ayuso et al., 2014; D’Costa et al., 2016). The SMA programme provides the clearest illustration, and one that DMD inherited along with the dose: the high dose reported as 2.0 × 10^14^ vg/kg in the phase 1 study corresponds to the 1.1 × 10^14^ vg/kg dose subsequently used in confirmatory trials and registration, the difference reflecting revalidation of the vector genome titration method rather than any change in the physical quantity of vector administered (Mendell et al., 2017; Day et al., 2021). Because DMD dose selection was anchored to these values (Section 4.1), the imprecision in the reference unit was inherited together with the dose, and cross-programme comparisons of the “same” dose in DMD rest on a foundation that was already assay-dependent. Nominally identical vg/kg doses across programs are therefore not strictly comparable, and apparent differences in potency or toxicity threshold between products may partly reflect assay methodology rather than biology. And trial design remains a limiting factor, particularly when short-term endpoints are applied to therapies that may act gradually as disease modifiers rather than producing rapid gains in strength (Fischer et al., 2024; Johnson et al., 2025). This is not merely a qualitative concern: as shown in Section 12.2, the sample sizes and 52-week windows used in pivotal DMD trials are, on plausible natural-history assumptions, insufficient to detect a clinically worthwhile slowing of decline, which means that a missed primary endpoint in these trials is weak evidence of absent effect.

## 7 The variable impact of exon skipping as a window onto therapeutic thresholds

### 7.1 Antisense oligonucleotide chemistries and the threshold question

Before AAV-microdystrophin reached the clinic, antisense oligonucleotide–mediated exon skipping provided the first sustained demonstration that dystrophin expression could be therapeutically modified in patients (Cirak et al., 2011; Mendell et al., 2013). Treating these drugs merely as a superseded stage is an oversimplification: exon skipping remains both a treatment modality and an informative biological experiment concerning how much dystrophin restoration is required for clinical effect.

The underlying concept is well defined. Many *DMD* mutations disrupt the reading frame; redirecting splicing to exclude a selected exon can restore the frame and permit synthesis of an internally shortened dystrophin analogous to that seen in Becker muscular dystrophy (Goyenvalle et al., 2004; Aartsma-Rus et al., 2009; Bladen et al., 2015). The approach is mutation-specific but applicable to substantial patient subsets, with exon 51 skipping historically among the broadest categories (Aartsma-Rus et al., 2009). Phosphorodiamidate morpholino oligomer (PMO) chemistry became the leading platform, leading to accelerated approval of eteplirsen, golodirsen, viltolarsen and casimersen for selected genotypes in the United States; the constructs, regimens, reported dystrophin levels and trial identifiers are set out in Table 3.

**TABLE 3:** Antisense and conjugate platforms evaluated in patients: chemistry, regimen, reported dystrophin restoration, and platform-specific toxicity.

Agent (INN/code; brand)	Chemistry	Target exon	Dose and route	Reported dystrophin (method)	Key trials (ID)	Status	Platform-specific toxicity
Drisapersen	2′-O-methyl phosphorothioate	51	6 mg/kg SC weekly	Variable, low	DEMAND II (NCT01153932); DEMAND III (NCT01254019)	FDA complete response letter 2016; discontinued	Injection-site reactions, thrombocytopenia, proteinuria/renal — attributable to PS backbone
Eteplirsen (EXONDYS 51)	PMO	51	30 mg/kg IV weekly	∼0.9% of normal (WB)	201/202 (NCT01396239/NCT01540409); PROMOVI (NCT02255552)	Accelerated approval 2016; PROMOVI did not confirm functional benefit	Generally well tolerated; port-related infections; hypersensitivity
Golodirsen (VYONDYS 53)	PMO	53	30 mg/kg IV weekly	0.10% → 1.02% (WB)	SKIP-NMD (NCT02310906); ESSENCE (NCT02500381)	Accelerated approval 2019 after initial CRL citing renal signal	Label warning for renal toxicity; infusion reactions
Viltolarsen (VILTEPSO)	PMO	53	80 mg/kg IV weekly	∼5.9% of normal (WB)	NCT02740972; RACER53 (NCT04060199)	Accelerated approval 2020; RACER53 reported not to meet primary endpoint	Renal monitoring advised; generally well tolerated
Casimersen (AMONDYS 45)	PMO	45	30 mg/kg IV weekly	0.93% → 1.74% (WB)	ESSENCE (NCT02500381)	Accelerated approval 2021; confirmation pending	As above
Vesleteplirsen (SRP-5051)	PPMO (cell-penetrating peptide–PMO)	51	30 mg/kg IV monthly	∼10-fold higher skipping than eteplirsen (sponsor)	MOMENTUM (NCT04004065)	FDA clinical hold 2022	Grade 3 hypomagnesaemia; hypokalaemia — renal tubular electrolyte wasting, the dose-limiting class toxicity
PGN-EDO51	PPMO (EDO peptide)	51	IV, escalating	Sponsor-reported skipping/dystrophin	CONNECT1/CONNECT2	Deprioritised	Hypomagnesaemia signal reported for the class
ENTR-601-44	EEV-conjugated PMO	44	IV	Sponsor-reported	Phase 1/2	Ongoing	Under evaluation
Delpacibart zotadirsen (del-zota; AOC 1044)	Anti-TfR1 mAb–PMO conjugate	44	IV, every 6 weeks	Substantially exceeding unconjugated PMOs; large CK reduction (sponsor + Etxaniz et al., 2025)	EXPLORE44/-OLE	Accelerated-approval submission planned	Infusion-related reactions; theoretical haematologic risk from TfR1 on erythroid precursors; ADA formation
Zeleciment rostudirsen (DYNE-251)	Anti-TfR1 Fab–PMO conjugate	51	20 mg/kg IV every 4 weeks (registrational cohort)	Low single-digit % of normal (sponsor)	DELIVER (NCT05524883)	Breakthrough Therapy Designation; topline December 2025	As above
WVE-N531	Stereopure PN-modified oligonucleotide	53	IV every 2 weeks	∼9% muscle-content-adjusted (sponsor)	FORWARD-53	Accelerated-approval pathway pursued	Under evaluation

ADA, anti-drug antibody; CRL, complete response letter; EEV, endosomal escape vehicle; PN, phosphoryl-guanidine; SC, subcutaneous; WB, Western blot. Entries drawn from peer-reviewed reports where available; values marked “sponsor” derive from company communications and are not peer reviewed. Dystrophin values are not comparable across programmes because normalisation, muscle-content adjustment and antibody/assay differ.

Two features of that record deserve more explicit statement than they usually receive. The first is that all four approvals were granted on a molecular surrogate rather than on function, and the reported surrogate values are extremely low: approximately 0.9% of normal for eteplirsen, an increase from 0.10% to 1.02% for golodirsen, from 0.93% to 1.74% for casimersen, and approximately 5.9% for viltolarsen (Charleston et al., 2018; Clemens et al., 2020; Frank et al., 2020). These values are not mutually comparable, because antibody, normalisation reference, muscle-content adjustment and quantification method differ between programmes—the point made forcefully during the eteplirsen debate (Aartsma-Rus and Arechavala-Gomeza, 2018). The practical consequence is that the ranking of these drugs by ‘dystrophin produced’ is largely an artefact of assay choice, and cross-programme comparison on that basis is not defensible. The second is that confirmatory functional evidence has not materialised: PROMOVI did not establish functional benefit for eteplirsen against a concurrent untreated cohort, and RACER53 has been reported not to meet its primary endpoint for viltolarsen, while ESSENCE remains the pivotal confirmation for golodirsen and casimersen. What supports continued use is largely long-term observational comparison with natural-history controls, showing delayed loss of ambulation and slower pulmonary decline in some treated populations (Mitelman et al., 2022; Iff et al., 2023; Iff et al., 2024) — evidence that is directionally consistent but confounded by treatment selection, steroid regimen and era of care.

The reason the levels are low is pharmacological rather than mechanistic, and it is instructive because it defines what the next-generation must fix. Unconjugated PMOs are uncharged and enter myofibres by a slow, non-receptor-mediated route; only a very small fraction of an administered dose reaches skeletal muscle, most is cleared renally within hours, and much of what does enter is retained in endosomes rather than reaching the nucleus (Moulton and Moulton, 2010; Gan et al., 2022). Because the target compartment is roughly a third of body mass, this delivery inefficiency cannot be overcome by dose escalation within a tolerable range. The chemistry is nonetheless the reason these drugs are usable at all: the phosphorodiamidate backbone is neutral and is not associated with the injection-site reactions, thrombocytopenia and proteinuria that ended development of the 2′-O-methyl phosphorothioate compound drisapersen ((Voit et al., 2014; Goemans et al., 2018) Table 3). Chemistry in this field therefore sets the safety ceiling, and delivery sets the efficacy ceiling; the two have proved difficult to raise simultaneously.

Discussion of these agents has often centered on absolute dystrophin levels, which are generally modest. The more informative point concerns therapeutic thresholds. Long-term observational studies have reported outcomes such as delayed loss of ambulation and slower respiratory decline relative to historical controls in some treated populations (Mitelman et al., 2022; Iff et al., 2023; Iff et al., 2024), while the overall magnitude of benefit has remained limited and robust phase III functional proof has been difficult to obtain (Voit et al., 2014; Goemans et al., 2018). On balance, exon skipping appears to occupy the lower portion of the dystrophin-restoration continuum: sufficient in some patients to delay loss of ambulation and slow the decline in forced vital capacity by a measurable interval, but not sufficient to shift the trajectory toward the Becker range. This position is scientifically useful because it is consistent with a nonlinear therapeutic relationship, in which a small increase in dystrophin can be meaningful yet substantial tissue stabilization may require crossing a higher threshold. Such a relationship would help explain both the persistence of modest benefit signals and the rationale for pursuing more potent approaches (Figure 4).

**FIGURE 4:**
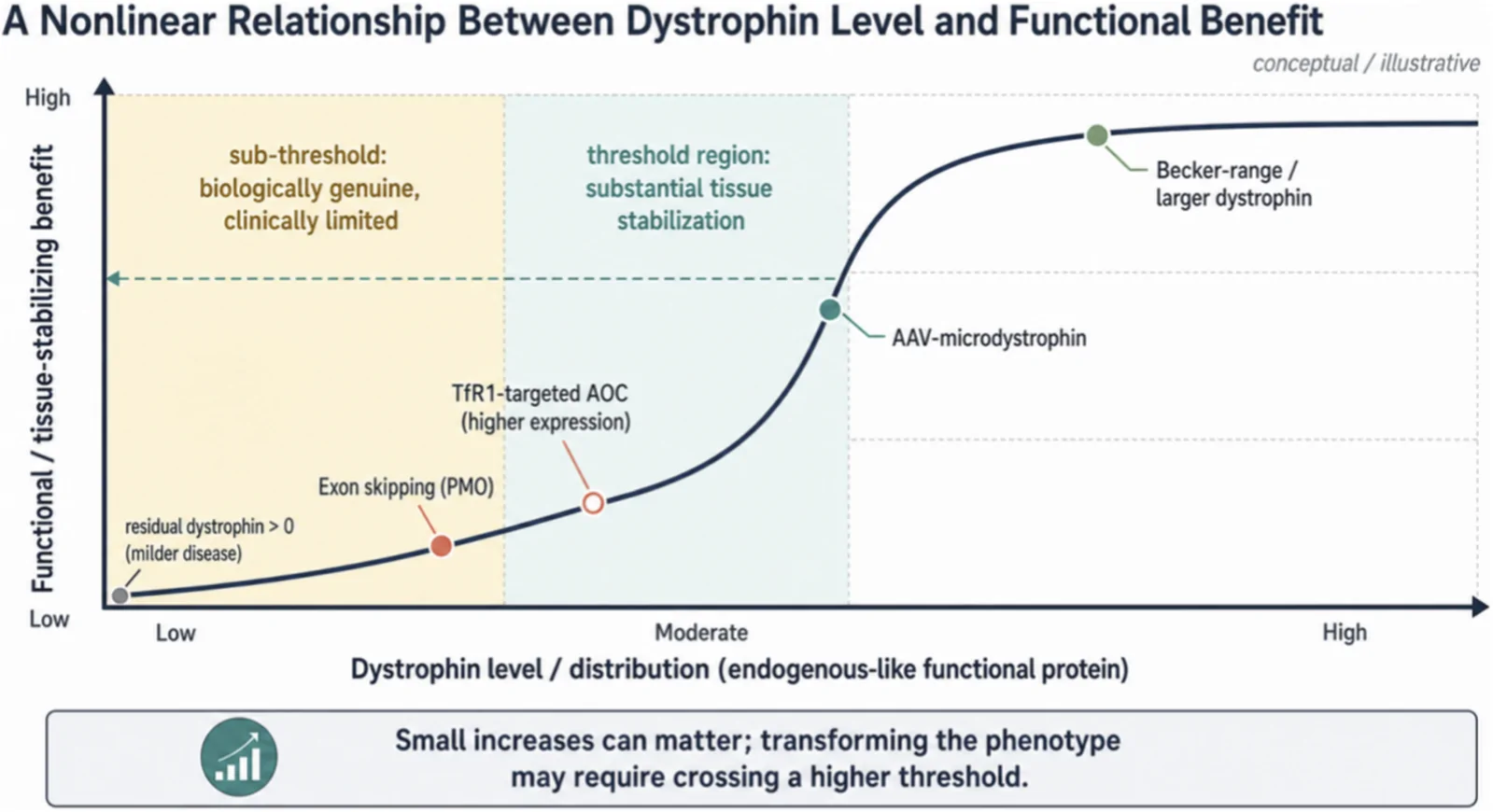
A conceptual nonlinear relationship between dystrophin restoration and clinical benefit. The schematic (illustrative, not quantitative) depicts benefit as a sigmoid function of the level and distribution of functional dystrophin. In the sub-threshold region (amber), restoration can be mechanistically genuine yet clinically modest—consistent with the position of conventional antisense exon skipping. Crossing a higher threshold region (teal) may be required for substantial tissue stabilization and phenotype modification, as suggested by Becker-range expression and larger-dystrophin strategies. Transferrin-receptor-targeted antibody–oligonucleotide conjugates are placed at an intermediate position, while AAV-microdystrophin is placed near the upper part of the threshold region; neither reaches the Becker-range plateau. This nonlinearity helps reconcile the persistence of modest benefit signals with the rationale for pursuing more potent, higher-magnitude approaches. U7-mediated correction in exon 2 duplications is the only current clinical strategy positioned at the wild-type rather than the Becker end of this axis, since both possible splicing outcomes yield either full-length 427-kDa dystrophin or the highly functional 423-kDa IRES-initiated proteoform.

Exon skipping also illustrates the importance of tissue distribution. Systemic PMOs can restore dystrophin in skeletal muscle, but cardiac activity has historically been weak, in part because of limited cellular uptake and endosomal sequestration (Moulton and Moulton, 2010; Gan et al., 2022; Lim et al., 2022). This is not a minor pharmacokinetic detail; it may define the ceiling of long-term benefit, since a therapy that slows skeletal decline without adequately protecting the myocardium leaves a major determinant of survival unaddressed. Peptide-conjugated PMOs and other next-generation chemistries are being developed to address this limitation, though toxicity concerns such as hypomagnesemia and potential renal injury have complicated progress (Desjardins et al., 2022; Aartsma-Rus, 2023). A more recent generation of transferrin receptor 1 (TfR1)-targeted antibody-oligonucleotide conjugates (AOCs) has produced the largest reported gains in systemic dystrophin restoration achieved by an antisense-based platform to date. Delpacibart zotadirsen (AOC 1044), which couples an exon 44-skipping PMO to an anti-TfR1 antibody, produced dystrophin expression increases substantially exceeding those historically reported for unconjugated PMOs, together with marked reductions in creatine kinase, in an early-phase trial in exon 44-skipping-amenable patients (Etxaniz et al., 2025). A comparable TfR1-targeting Fab-PMO conjugate directed at exon 51, zeleciment rostudirsen (formerly DYNE-251), received FDA Breakthrough Therapy Designation and reported topline data from its registrational expansion cohort in December 2025, in support of a planned submission for U.S. accelerated approval-Dyne Therapeutics 2025. These results suggest that TfR1-mediated muscle targeting may substantially narrow the gap between the “sufficient but modest” restoration historically associated with exon skipping and the higher-magnitude expression sought from gene-transfer approaches, though cardiac penetration, long-term functional benefit, and durability of this conjugate strategy remain to be established in larger and longer trials.

### 7.2 Improving delivery: peptide conjugates, receptor-targeted conjugates, and the toxicities they introduce

If the limitation of unconjugated PMOs is uptake, the logical response is to give the oligonucleotide an active route into muscle. Three approaches have reached patients, and each has traded the benign safety profile of the parent chemistry for greater potency.

Peptide-conjugated PMOs (PPMOs). Arginine-rich cell-penetrating peptides increase myofibre and, importantly, cardiomyocyte uptake, and the Pip6 series produced substantially greater cardiac exon skipping than unconjugated PMO in preclinical models (Betts et al., 2012). Clinical translation has been constrained by a specific and reproducible toxicity. Vesleteplirsen (SRP-5051) generated approximately tenfold greater skipping than eteplirsen but caused grade 3 hypomagnesaemia and hypokalaemia, consistent with cationic peptide accumulation in the proximal tubule and consequent renal electrolyte wasting; the programme was placed on clinical hold in 2022 and subsequently discontinued. A comparable hypomagnesaemia signal has been reported for other peptide-conjugated programmes (Table 3). The lesson is not that peptide conjugation is unworkable but that the therapeutic window of a PPMO is set in the kidney rather than in muscle, and that magnesium and potassium monitoring, and renal tubular biomarkers, are the relevant safety instruments. Alternative peptides—DG9 and endosomal-escape designs—are being pursued explicitly to widen that window (Desjardins et al., 2022; Lim et al., 2022).

Transferrin receptor 1–targeted conjugates. TfR1 is highly expressed on skeletal and cardiac muscle and internalises rapidly, which makes it an attractive route (Desjardins et al., 2022). Two clinical constructs exploit it with different architectures: delpacibart zotadirsen (AOC 1044) couples an exon 44–skipping PMO to a full anti-TfR1 monoclonal antibody, and zeleciment rostudirsen (formerly DYNE-251) couples an exon 51–skipping PMO to an anti-TfR1 Fab. The distinction is not cosmetic: a full antibody offers a longer circulating half-life and less frequent dosing, whereas a Fab has lower molecular weight, potentially better tissue penetration, and no Fc-mediated effector engagement. Reported dystrophin restoration substantially exceeds that historically achieved with unconjugated PMOs, together with marked creatine kinase reduction ((Etxaniz et al., 2025) Table 3), and both programmes are being advanced toward accelerated approval.

Three cautions apply, and they follow directly from the framework of this review. First, TfR1 is not muscle-specific; it is constitutively expressed on erythroid precursors and at the blood–brain barrier, so haematologic monitoring is a rational class requirement even where it has not yet been necessary clinically, and receptor-mediated targeting to muscle is inherently a matter of relative rather than absolute selectivity. Second, these are repeat-administered biologics, so anti-drug antibodies and infusion reactions are foreseeable long-term issues that a one-time vector does not have. Third, and most importantly for interpretation, the reported dystrophin levels are the strongest achieved by any antisense platform, yet the same evidentiary problem recurs: they are biopsy measurements in accessible skeletal muscle at a single timepoint, cardiac and diaphragmatic penetration is not directly demonstrated in patients, and no functional confirmation is yet available. Whether TfR1 targeting has genuinely moved exon skipping across the threshold region of Figure 4, or has moved the surrogate without moving the trajectory, is exactly the question that the endpoint framework proposed in Section 12 is intended to answer, and it should not be settled by expression data alone.

### 7.3 U7 snRNA–mediated splice correction and DMD exon 2 duplications: the field’s only route to full-length dystrophin

Every strategy discussed so far accepts a truncated protein. Exon skipping generates internally deleted pseudodystrophins by design, and single-vector microdystrophins are truncated because the AAV packaging limit requires it Section 4. One clinical programme is exempt from this constraint, and because it bears directly on the question of whether truncation matters—the question raised in Section 3.2 and left open there—it warrants separate treatment. We follow here the framework set out by Roberts (2023).

A genotype in which both splicing outcomes are therapeutic. Whole-exon duplications account for approximately 11% of disease-causing DMD variants, and duplication of exon 2 constitutes roughly 10% of these, making it the most frequently duplicated single exon and affecting on the order of 1% of all patients (Bladen et al., 2015; Roberts, 2023). Splice-switching approaches cannot distinguish the original exon from its duplicate, so treatment yields either single- or double-exon-2 skipping, and in most duplication genotypes this unpredictability would be disabling. Exon 2 is the exception, because both outcomes are corrective. Removal of one copy regenerates a wild-type transcript and therefore entirely normal full-length 427-kDa dystrophin. Removal of both copies causes premature termination in exon 3, but an internal ribosome entry site in exon 5 then drives cap-independent translation from an alternative start codon in exon 6, producing a 423-kDa N-terminally truncated proteoform lacking part of the first actin-binding domain; this proteoform is highly functional and has been associated with a very mild Becker phenotype in which ambulation was maintained into at least the seventh decade (Wein et al., 2014; Roberts, 2023). A mechanistic detail explains why the second product appears only after therapy: the exon 2 duplication itself inactivates the exon 5 IRES, so cap-independent initiation is not available in the untreated state and is restored only once the duplicated region is removed (Wein et al., 2014). The genotype is therefore unusually forgiving of an inherently imprecise intervention—a rare instance in DMD in which the biology absorbs, rather than amplifies, the imprecision of the tool.

The construct and the U7 rationale. The clinical vector, scAAV9.U7.ACCA, is a self-complementary AAV9 genome encoding four modified U7 small nuclear RNA antisense cassettes directed at the exon 2 splice acceptor and donor sites (Roberts, 2023). U7 snRNA is not a spliceosomal component—natively it directs histone pre-mRNA 3′-end processing—but replacing its histone-downstream-element–binding sequence with an antisense sequence, and substituting the optimised SmOPT Sm-binding site, converts it into a nuclear-retained, Sm-class snRNP that acts as a stable, continuously expressed splice-modulating agent (Gorman et al., 1998; Schümperli and Pillai, 2004). Two properties follow that are directly relevant to the constraints set out in this review. The cassette is small enough—a few hundred base pairs, transcribed from its own natural promoter with no exogenous regulatory elements required—that four independent antisense units fit within a self-complementary genome, which is not possible for any protein-coding cargo. And the antisense agent is produced continuously from the transduced nucleus, so a single administration substitutes for the indefinite repeat dosing that synthetic oligonucleotides require.

Efficacy, and where it is highest. The programme’s preclinical foundation includes proof of concept, dose finding, off-target splicing analysis, and non-human primate toxicology (Simmons et al., 2021). The most informative recent addition is a long-term study in Dup2 mice treated intravenously at 3 months of age with 3 × 10^13^ vg/kg—a previously defined minimally efficacious dose—and analysed 18 months later (Roberts, 2023). Therapeutically skipped transcripts were most abundant in heart (73%), followed by tibialis anterior (46%) and diaphragm (32%), and dystrophin protein followed the same rank order at 65%, 42% and 18% of wild-type levels respectively. Force output improved in tibialis anterior and diaphragm without reaching wild-type values, eccentric contraction–induced force was partially rescued, and mean myofibre diameter normalised, although central nucleation was slightly increased after treatment. Critically, the degree of functional correction at 18 months resembled that reported previously at three and 6 months, indicating sustained rather than waning partial rescue (Roberts, 2023).

Two features of this distribution profile deserve emphasis because they cut in opposite directions and are rarely commented on together. The first is that restoration was highest in the heart. This is the inverse of the pattern that limits systemic phosphorodiamidate morpholino oligomers, whose weak cardiac activity we argued in Section 7.1 may define the ceiling of their long-term benefit, and it indicates that the cardiac deficiency of exon skipping is a property of oligonucleotide pharmacokinetics rather than of splice modulation as a mechanism. The second is that the diaphragm was the least well corrected tissue, at 18% of wild-type protein—the lowest of the three sampled compartments. Since respiratory failure remains a principal cause of death in DMD, and since the diaphragm is not accessible to biopsy in patients, this is not a minor observation: it means that the tissue with the clearest bearing on survival was, in the one model where all compartments can be measured directly, the one least well served. Both observations are consistent with the general argument of Section 3.3 that coverage is compartment-specific and that a single skeletal-muscle readout systematically misrepresents systemic adequacy.

Clinical status, and the first full-length dystrophin in a patient. The vector has progressed to a first-in-human phase I/II systemic trial in boys with exon 2 duplications (NCT04240314). Preliminary, non-peer-reviewed reports describe three treated patients with dystrophin at approximately 70% of normal levels and 99% of myofibres dystrophin-positive on 4-month post-treatment biopsy (Roberts, 2023). If confirmed, this is the first gene therapy to produce full-length dystrophin in DMD patient muscle. Two qualifications are essential. These data are not peer reviewed, the cohort comprises three patients, and—as we argue throughout—a 4-month biopsy value in one accessible skeletal muscle is precisely the kind of readout whose relationship to clinical benefit is not linear (Section 3.1, Section 3.3). Nor does an expression value of this magnitude establish that full-length protein is superior, since it is confounded with the level achieved.

Why this small programme has disproportionate interpretive value. Three points extend beyond the roughly 1% of patients eligible for it.

First, it constitutes the field’s only clinical test of whether restoring full-length dystrophin is materially better than restoring a truncated surrogate. In Section 3.2 we argued that constructs are not interchangeable, that the omission of repeats R16–R17 predicts persistent loss of nNOS-dependent vasodilatation, and that larger dystrophins merit direct comparison rather than dismissal on packaging grounds. That comparison cannot be performed within the microdystrophin class, because all clinical constructs omit the same regions. The exon 2 duplication cohort supplies, by accident of genotype rather than by design, a group in which the protein is complete. If long-term follow-up in these patients shows preservation of function—particularly exercise-dependent perfusion, diaphragmatic force and left-ventricular systolic function—that exceeds what microdystrophin programmes achieve, the case for larger dystrophins and multi-vector reconstitution strategies (Section 10.2) is strengthened considerably; if it does not, the truncation trade-off is vindicated. Either result is informative, and the field currently has no other way of obtaining it. We would add one caveat that this framing usually omits: because the two comparisons differ simultaneously in construct, capsid, dose and patient genotype, any such comparison will be indirect, and it should be pre-specified and matched rather than assembled retrospectively.

Second, the dose is instructive. At 3 × 10^13^ vg/kg the effective dose in the Dup2 study is roughly an order of magnitude below the 1–2 × 10^14^ vg/kg range used across systemic microdystrophin programmes (Roberts, 2023). This is directly relevant to the argument of Section 4.1: the systemic dose in DMD was fixed by dose-escalation in mdx mice within an upper bound inherited from spinal muscular atrophy, rather than by ascending dose-finding in patients, with the consequence that the therapeutic window has never been defined at its lower edge in humans. Together with GNT0004 (Section 6), the U7 programme is one of only two DMD efforts operating substantially below that inherited ceiling, and it does so with a cargo that is small enough to permit high genome copy numbers per particle in a self-complementary configuration. The implication is not that microdystrophin doses could simply be reduced tenfold, since cargo, promoter and expression requirements differ, but that the inherited ceiling is not a physical requirement of AAV therapy in DMD and that the region below it remains largely unexplored.

Third, the immunological position of the platform is distinctive, and for a structural reason. Section 8.2 identifies transgene-directed immunity as the most consequential recent safety finding in this field: patients whose deletions remove the exons encoding epitopes present in a therapeutic construct have never been tolerized to those epitopes, and severe immune-mediated myositis has followed (Bönnemann et al., 2023; Potter et al., 2025). A U7 vector encodes no protein at all—only a small nuclear RNA—and in the exon 2 duplication setting the protein that appears at the sarcolemma is the patient’s own wild-type dystrophin, so no novel junctional epitopes are created. What remains is anti-capsid immunity, common to every AAV platform, together with any response to residual revertant-primed dystrophin-reactive T cells. The platform therefore dissociates two liabilities that are usually bundled, and it demonstrates that transgene immunogenicity is a property of expressing a designed dystrophin surrogate rather than an inevitable consequence of AAV-mediated dystrophin restoration.

The residual constraint is persistence, and the mouse data do not fully resolve it. The limitation the approach does not escape is the one identified in Section 3.4. The corrective agent is encoded on an episomal genome, and expression can decline through three distinct routes: dilution as new myonuclei accrete during growth, physical loss of genomes from regions undergoing necrosis, and functional silencing through chromatin modification and cytosine methylation of the viral DNA (Roberts, 2023). Direct loss of AAV genomes from dystrophic muscle has been demonstrated for U7 constructs (Le Hir et al., 2013), although functional recovery was maintained over an extended period in the golden retriever model. The 18-month persistence in Dup2 mice is genuinely encouraging, but its extrapolation is limited in a way that matters for exactly the reason set out in Section 2: the mice were dosed at 3 months of age, when growth is largely complete, whereas a child dosed at four or 5 years will undergo a several-fold increase in muscle mass and a corresponding accretion of myonuclei that were never transduced. Eighteen months in a largely grown mouse therefore does not establish durability across the one to two decades over which the disease progresses in a growing child, and because AAV redosing is currently precluded (Section 9.4) this is the question on which long-term follow-up of NCT04240314 will be most informative.

Timing. Preliminary trial observations suggest that the effectiveness of dystrophin restoration is inversely related to age at treatment, and one participant is the youngest patient yet to receive gene therapy for DMD (Roberts, 2023). This is consistent with the tissue-state argument of Section 2 — that the same construct at the same dose is not the same intervention at 4 years and at twelve—and it reinforces the case for earlier identification through newborn screening (Section 11), while also illustrating the point acknowledged in the screening evidence review that optimal treatment timing has not been established.

Position within the framework of this review. Referred to the four dimensions of Figure 1 U7-mediated correction in exon 2 duplications is the only current platform that fully satisfies the structural dimension—a complete protein retaining every domain, including R16–R17 and the C-terminal syntrophin/dystrobrevin-binding region that clinical microdystrophins omit—while achieving high amount, broad coverage including the myocardium, and doing so at a fraction of the vector dose and without introducing an antigenic protein. Its exposure is concentrated almost entirely on persistence, and on the very small population it can serve. That profile is close to the mirror image of AAV-microdystrophin, which achieves broad coverage of a structurally incomplete and potentially antigenic protein at a dose near the ceiling of tolerability. The juxtaposition makes the central claim of this review unusually legible: no single dimension is the bottleneck, and progress has consisted of trading one constraint for another rather than removing any of them.

### 7.4 Strategic position of splice modulation relative to gene transfer

Strategically, exon skipping is best viewed as complementary to gene transfer rather than as a direct competitor (Aartsma-Rus and Corey, 2020). It preserves endogenous gene regulation, avoids expression of an exogenous dystrophin analog, and in some contexts may be more compatible with repeated administration than AAV-based platforms (Duan et al., 2021). Its principal limitations are mutation specificity, modest protein restoration, and limited cardiac efficacy (Aartsma-Rus et al., 2017). A plausible future is one in which lower-risk chronic correction and higher-intensity one-time gene delivery serve different patients or disease stages rather than one approach simply replacing the other. Whatever the eventual balance, exon skipping has already established one clinically relevant point: partial dystrophin restoration, even when far from complete, is not biologically trivial—an observation that remains foundational for the strategies that followed.

## 8 Corticosteroids and non-dystrophin pharmacotherapy: the substrate, the comparator, and the confounder

Discussions of dystrophin-restoring therapy tend to treat pharmacological management as background. This is a mistake for three distinct reasons, and each bears on how gene- and oligonucleotide-based therapies should be designed and evaluated. Approved and late-stage non-dystrophin agents are summarised in Table 4.

**TABLE 4:** Approved and late-stage non-dystrophin pharmacotherapy in DMD.

Drug (brand)	Class/mechanism	Regimen	Pivotal evidence (ID)	Regulatory status	Principal adverse effects	Critical appraisal
Prednisone/prednisolone	Glucocorticoid receptor agonist	0.75 mg/kg/day	FOR-DMD (NCT01603407) (Guglieri et al., 2022)	Standard of care; not DMD-labelled in most jurisdictions	Weight gain, growth suppression, BMD loss/fracture, behavioural change, adrenal suppression	Daily regimens superior to intermittent; the only intervention with unambiguous effect on ambulation duration, yet never placebo-controlled to modern standards
Deflazacort (EMFLAZA)	Glucocorticoid	0.9 mg/kg/day	Griggs et al., 2016; FOR-DMD	FDA approved 2017	Less weight gain than prednisone; more cataracts	Approval rested substantially on a decades-old dataset; cost–benefit versus prednisone contested
Vamorolone (AGAMREE)	Dissociative steroidal GR modulator/MR antagonist (Δ9,11)	6 mg/kg/day	VISION-DMD (NCT03439670); Dang et al. (2024)	FDA approved 2023; EU approved	Cushingoid features, weight gain, adrenal suppression requiring stress dosing, vitamin D deficiency	Non-inferior to prednisone on TTSTAND velocity with less growth stunting and less BMD loss — the clearest demonstration in DMD that a safety-differentiated comparator trial can succeed where superiority trials fail
Givinostat (DUVYZAT)	HDAC inhibitor; anti-fibrotic/pro-regenerative	Weight-banded oral, twice daily	EPIDYS (NCT02851797); Mercuri et al. (2024)	FDA approved 2024 (≥6 years, all genotypes)	Thrombocytopenia (dose-limiting, requires monitoring), diarrhoea, abdominal pain, hypertriglyceridaemia	First mutation-agnostic disease-modifier approved on a functional endpoint (four-stair climb); mechanistically complementary to dystrophin restoration since it targets the substrate rather than the protein
Ataluren (TRANSLARNA)	Nonsense-mutation readthrough	40 mg/kg/day oral	ACT DMD (NCT01826487); Study 041 (NCT03179631)	EMA conditional approval 2014	GI, renal, hypertension	Cautionary case: primary endpoint missed, benefit rested on a *post hoc* 300–400 m 6MWD subgroup, confirmation failed — the clearest illustration of why *post hoc* subgroups cannot substitute for pre-specified endpoints
Idebenone	Antioxidant/mitochondrial	Oral	DELOS; SIDEROS	Not approved for DMD	—	Respiratory signal not confirmed in the larger trial
Pamrevlumab	Anti-CTGF; anti-fibrotic	IV	LELANTOS-1/-2	Development discontinued	—	Anti-fibrotic rationale sound; endpoints and stage selection remain the plausible failure point
Edasalonexent	NF-κB inhibitor	Oral	PolarisDMD	Discontinued	—	As above
Sevasemten (EDG-5506)	Fast-myosin modulator	Oral	GRAND CANYON (BMD); DMD cohorts	Investigational	—	Aims at contraction-induced injury rather than dystrophin; would act on the same substrate that gene therapy enters
Eplerenone/ACE inhibitors, β-blockers	Cardioprotective	Standard cardiology dosing	Raman et al. (2015)	Standard of care	Hyperkalaemia; hypotension	Establish the cardiac background against which any gene-therapy cardiac benefit must be demonstrated

BMD, bone mineral density; GR, glucocorticoid receptor; MR, mineralocorticoid receptor; TTSTAND, time to stand from supine.

### 8.1 Corticosteroids: the benchmark that dystrophin restoration has not yet displaced

Glucocorticoids remain the only intervention with an unambiguous effect on the duration of ambulation. FOR-DMD compared daily prednisone, intermittent prednisone and daily deflazacort and established that daily regimens are superior to intermittent dosing, providing the first modern randomised basis for a practice adopted decades earlier (Guglieri et al., 2022); deflazacort was licensed in the United States in 2017 on a dataset that included trials conducted in the 1990s (Griggs et al., 2016). Two implications follow. First, corticosteroids define the effect size that a disease-modifying therapy must exceed to be clinically meaningful, and because essentially all trial participants are steroid-treated, gene therapy is always evaluated as an add-on to an active background therapy—which compresses the achievable incremental effect and is rarely acknowledged when a missed endpoint is interpreted. Second, chronic steroid exposure alters the very tissue and host that a vector encounters: it modifies inflammatory tone, fibrotic remodelling, growth and bone, and it constitutes chronic immunosuppression, which is one reason the tolerability margin observed in steroid-naive SMA infants had no strong reason to transfer to DMD (Section 4.1).

Vamorolone is instructive beyond its own indication. It is a dissociative steroidal compound that retains glucocorticoid-receptor–mediated anti-inflammatory activity while antagonising the mineralocorticoid receptor, and in VISION-DMD it was non-inferior to prednisone 0.75 mg/kg/day on time-to-stand velocity with less growth suppression and less loss of bone mineral density, although adrenal suppression still requires stress dosing (Dang et al., 2024; Table 4). Methodologically, this is the clearest recent example in DMD of a trial that succeeded because it was designed to demonstrate safety differentiation against an active comparator rather than superiority against decline—a design consideration we return to in Section 12.

### 8.2 Non-steroidal disease modifiers act on the substrate that gene therapy must enter

Givinostat, a histone deacetylase inhibitor with anti-fibrotic and pro-regenerative activity, was approved in 2024 on the basis of EPIDYS, in which the primary endpoint was change in time to climb four stairs and the effect favoured treatment in a mutation-agnostic population; thrombocytopenia is dose-limiting and requires platelet monitoring (Bettica et al., 2016; Mercuri et al., 2024). This matters for the argument of Section 2. If fibro-fatty replacement and impaired regeneration determine how much benefit a given level of dystrophin can produce, then an agent that improves the substrate is not a competitor to gene therapy but a potential enabler of it, and the sequencing and possible interaction of the two is an empirical question that no current trial is designed to answer. The same reasoning applies to anti-fibrotic and myosin-modulating strategies, and to standard cardioprotective therapy, which defines the cardiac background against which any vector-mediated cardiac benefit would have to be demonstrated (Raman et al., 2015; Section 10.1).

### 8.3 Ataluren as the field’s clearest cautionary precedent

Ataluren received conditional EMA approval for nonsense-mutation DMD in 2014 despite a phase 2b trial in which the primary endpoint was not met; the therapeutic case rested substantially on a *post hoc* subgroup defined by baseline 6-min walk distance, the confirmatory Study 041 did not succeed, and the marketing authorisation was ultimately not renewed (Bushby et al., 2014; McDonald et al., 2017; Table 4). We include this history deliberately, because the interpretive pattern—a plausible mechanism, a missed primary endpoint, a favourable *post hoc* subgroup, prolonged conditional access, and eventual non-confirmation—is precisely the pattern now being debated for AAV-microdystrophin (Section 6). The relevant conclusion is not that conditional approval is illegitimate, but that the field has already run this experiment once and knows the outcome when confirmatory endpoints are not pre-specified and adequately powered. That is the substantive motivation for the endpoint criteria proposed in Section 12.

## 9 Immunobiology as a principal constraint on AAV-based therapy

A recurring theme in recent years is that immune biology is not a peripheral issue but a principal factor determining what current AAV-based therapies can safely achieve. This operates at several levels: pre-existing antibodies to the capsid, innate responses to systemic vector exposure, complement activation, hepatotoxicity, and adaptive responses to the transgene itself (Figure 5). It is equally important, however, not to treat these constraints as fixed. Each of them is now the object of active mitigation strategies, and the practical question is no longer whether anti-AAV immunity limits therapy—it plainly does—but how much of that limitation is modifiable, and at what cost.

**FIGURE 5:**
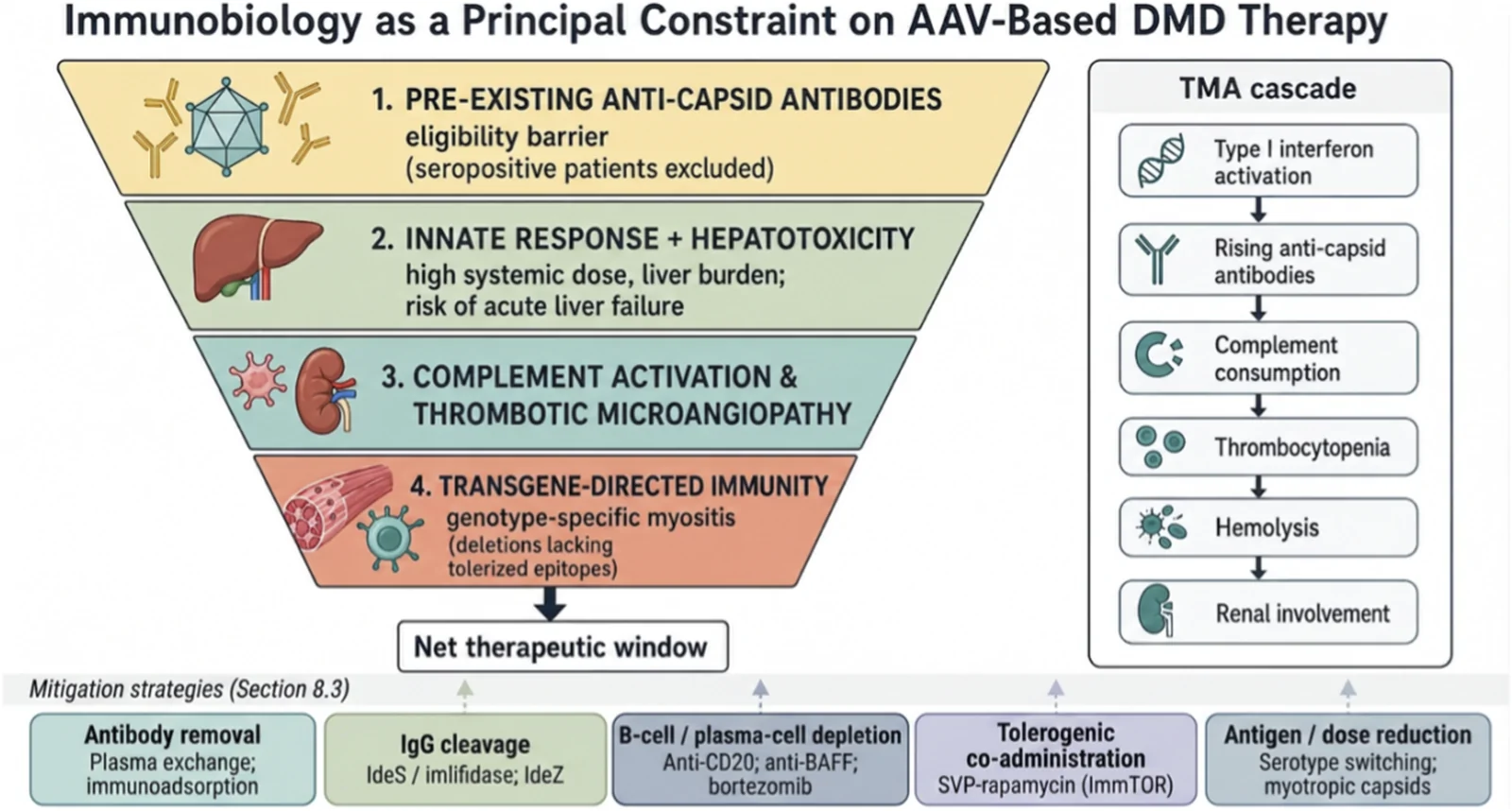
Immunobiology constrains AAV-based dystrophin restoration at multiple, sequential levels. Pre-existing anti-capsid antibodies first restrict eligibility (top band). Systemic high-dose delivery then imposes an innate and hepatic burden, including a risk of acute liver failure that prompted regulatory boxed-warning labeling. Complement activation can precipitate thrombotic microangiopathy through a defined cascade (right inset: type I interferon activation→rising anti-capsid antibodies→complement consumption→thrombocytopenia→hemolysis→renal involvement). Finally, transgene-directed immunity produces genotype-specific myositis in patients whose deletions leave them untolerized to epitopes encoded by the therapeutic construct. Because each layer narrows the net therapeutic window, safety and efficacy are not fully separable; a useful measure of progress may be the ratio of corrected muscle mass to total immunological burden. A lower band summarises the corresponding mitigation strategies now under development at each level—apheresis and capsid-selective immunoadsorption and IgG-cleaving endopeptidases acting on pre-existing antibody; dose reduction through myotropic capsid engineering acting on innate and hepatic burden; B-cell/plasma-cell depletion and tolerogenic nanoparticles acting on the anamnestic response that currently precludes re-administration; and genotype-aware patient selection and epitope-aware transgene design acting on transgene-directed immunity. These act at different levels and are only partially additive, which is why none individually converts AAV into a redosable platform.

### 9.1 Pre-existing humoral immunity, innate activation, and hepatic risk

The first barrier is eligibility. Neutralizing antibodies to commonly used AAV serotypes are prevalent in the general population and in boys with DMD, with estimates varying by serotype but often high enough to exclude a meaningful fraction of otherwise suitable patients (Leborgne et al., 2019; Verma et al., 2023). A therapy available only to seronegative individuals is not fully universal even before cost and access are considered, and immunological history, rather than clinical need alone, becomes a determinant of eligibility.

Pre-existing antibodies are only the starting point. Systemic infusion of high-dose AAV exposes the host to a large burden of capsid protein and vector genomes, much of which traffics through the liver; hepatotoxicity is consequently among the most common clinically significant adverse events (Costa Verdera et al., 2020; Kishimoto and Samulski, 2022). Many patients develop transient transaminase elevations that can be managed with corticosteroids or intensified immunosuppression (Wagner et al., 2021; Oskoui et al., 2025). However, hepatotoxicity can no longer be described as uniformly manageable: fatal acute liver failure has occurred, particularly in more vulnerable non-ambulatory patients receiving systemic microdystrophin gene therapy (Oskoui et al., 2025; Taylor, 2025). This risk has since been formalized at the regulatory level. Following additional reports of fatal acute liver failure in non-ambulatory boys treated with delandistrogene moxeparvovec, the U.S. Food and Drug Administration approved a revised label in November 2025 that added a boxed warning—its highest-level safety designation—for acute serious liver injury and acute liver failure, and simultaneously narrowed the approved indication to ambulatory patients aged 4 years and older. The revised labeling also mandates weekly liver function monitoring for at least 3 months post-infusion and excludes patients with deletions spanning exons 8 and/or 9 of the DMD gene. This regulatory narrowing—from a broader ambulatory-and-non-ambulatory population toward a younger, less advanced disease stage—provides a concrete, real-world illustration of this review’s central claim that immunological and hepatic tolerability, disease stage, and efficacy cannot be evaluated independently (Section 2; Section 9.3). This affects both risk–benefit assessment and the need for more specific biomarkers, such as glutamate dehydrogenase for hepatocellular injury (Wagner et al., 2021).

Complement activation and thrombotic microangiopathy (TMA) constitute another important axis of risk. Detailed analysis of TMA cases in the Pfizer program described a plausible sequence involving type I interferon activation, rising anti-capsid antibodies, complement consumption, thrombocytopenia, hemolysis, and renal involvement (Costa-Verdera et al., 2023; Chamberlain et al., 2025). Such events are concerning because they suggest that toxicity may arise from dose-dependent activation of innate immune circuits in susceptible individuals rather than being purely idiosyncratic. If so, the relevant question is not only whether steroids can suppress toxicity, but whether current vectors require an immunological burden for body-wide delivery that makes a degree of toxicity difficult to separate from the platform.

### 9.2 Transgene-directed immunity as a genotype-specific liability

The most consequential immune finding in recent years concerns the transgene itself. The possibility that newly expressed dystrophin or microdystrophin might be recognized as foreign in patients lacking endogenous exposure had long been acknowledged but was incompletely characterized until a cross-trial investigation of severe myositis cases (Bönnemann et al., 2023). Patients treated in different trials with different vectors and promoters developed strikingly similar severe adverse events several weeks after dosing, including profound myositis, loss of ambulation, bulbar and respiratory weakness, and, in some cases, myocarditis. The cardiac component of these events is itself instructive for monitoring. Because myocardial involvement cannot be biopsied in this setting, its detection depends on the same non-invasive tools discussed above—serial hs-cTnI, which rises acutely with myocyte injury, and CMR with T2 mapping and late gadolinium enhancement, which distinguish acute edema from established fibrosis (Messroghli et al., 2017; Voleti et al., 2020). Systematic troponin surveillance in the fordadistrogene movaparvovec program illustrates both the feasibility and the necessity of this approach in the peri-infusion period (Sherlock et al., 2025). Cardiac biomarker and imaging surveillance should therefore be regarded as a safety requirement of systemic AAV administration in DMD, not only as an efficacy measure. The shared feature was genetic: affected patients carried deletions spanning a region of the *DMD* gene, while the therapeutic constructs contained epitopes encoded by exons to which these patients had never been immunologically tolerized (Bönnemann et al., 2023; Servais et al., 2023). This converted a general concern into a concrete, mutation-specific risk factor.

Importantly, dystrophin immunogenicity may not be determined solely by the relationship between the patient’s deletion boundaries and the therapeutic construct. Pre-existing dystrophin-reactive T cells, prior immune priming by revertant fibers, chronic inflammation within dystrophic muscle, HLA-dependent epitope presentation, antigen cross-reactivity, and novel junctional epitopes created by truncated dystrophin constructs may also influence the risk and severity of immune responses. These considerations support prospective assessment of anti-dystrophin cellular immunity, together with genotype- and potentially HLA-informed risk stratification. They also provide a rationale for epitope-aware transgene design and tolerogenic protocols, particularly in older patients who may already have established immune memory against dystrophin-related antigens (Górecki et al., 2025).

Several implications follow. Transgene immunogenicity should be incorporated into patient selection as rigorously as anti-capsid serology. A single microdystrophin construct cannot be assumed to be equally suitable across genotypes, since the same construct may be relatively safe in one genotype and hazardous in another. And transgene design may need to evolve not only for functional performance but also to reduce immunogenicity, whether through epitope-aware redesign, alternative domain configurations, or paralog-based strategies such as microutrophin (Kennedy et al., 2018; Song et al., 2019; Wasala et al., 2023).

It follows that this liability is specific to platforms encoding a dystrophin surrogate. Splice-modulating strategies that restore the endogenous transcript—including AAV-U7 approaches, which express only a small nuclear RNA and, in exon 2 duplications, yield wild-type protein—are exposed to anti-capsid but not to construct-derived transgene immunity (Section 7.2).

### 9.3 Emerging strategies for managing pre-existing immunity and enabling vector re-administration

The account given above describes anti-AAV immunity largely as a boundary condition: seropositive patients are excluded, redosing is unavailable, and the antigenic burden of systemic delivery is simply accepted. That description is increasingly incomplete. Over the past decade, pre-existing neutralizing antibodies and the single-use constraint have been reframed from absolute exclusions into partially modifiable variables, and several mitigation strategies now span the range from preclinical proof of concept to clinical use. Because DMD requires among the highest systemic vector doses of any indication, and because eligibility restrictions bear directly on equity of access, these approaches are of particular relevance here—even though most were developed in liver-directed settings and their transferability to body-wide muscle transduction should not be assumed.

Physical removal of circulating antibodies. The most direct approach is depletion of anti-capsid IgG before infusion. Therapeutic plasma exchange (TPE) reduces neutralizing titers, and repeated cycles can convert previously ineligible individuals to titers compatible with transduction (Monteilhet et al., 2011). Its relevance to DMD specifically was established early: in seropositive non-human primates, plasmapheresis before vascular delivery restored microdystrophin expression that pre-existing antibodies would otherwise have abolished (Chicoine et al., 2014), and more recent work has extended this to AAVrh74, the capsid used in an approved DMD product (Potter et al., 2024). Capsid-selective immunoadsorption represents a refinement, removing anti-AAV IgG on an affinity matrix while largely preserving total immunoglobulin and thereby limiting the infection risk of non-selective exchange (Bertin et al., 2020; Orlowski et al., 2020); in non-human primates this approach has enabled repeated hepatic delivery of the same serotype (Salas et al., 2019). The limitations here are practical rather than conceptual. TPE and immunoadsorption are resource-intensive, require adequate vascular access in young children, achieve only partial reduction when starting titers are high, and—critically—do nothing to prevent the anamnestic antibody response that follows vector exposure.

Enzymatic cleavage of IgG. A pharmacological alternative is enzymatic degradation of circulating IgG. Bacterial endopeptidases of the IdeS family, derived from *Streptococcus* pyogenes and related organisms, cleave IgG at the lower hinge to yield F(ab')_2_ and Fc fragments, abolishing neutralizing and effector function within hours. Imlifidase (IdeS) is already clinically validated in a different context, having enabled transplantation in highly sensitized recipients (Jordan et al., 2017). In gene therapy models, IdeS and the related IdeZ restored liver and muscle transduction in seropositive non-human primates and permitted vector re-administration in previously dosed mice (Leborgne et al., 2020), and independent work confirmed rescue of AAV transduction in the presence of neutralizing antibodies (Elmore et al., 2020). The appeal relative to apheresis is logistical: administration is rapid, does not require extracorporeal circuits, and can in principle be repeated. The constraints are equally clear. The effect is transient, with IgG recovering over one to 2 weeks; pre-existing anti-IdeS antibodies are common in the population and may blunt activity; and the enzyme itself is immunogenic, which complicates repeated use. Next-generation and less immunogenic endopeptidases are in development, and clinical evaluation of IgG protease pre-treatment as an enabling step for systemic microdystrophin gene therapy in seropositive DMD patients has begun.

B-cell and plasma-cell directed regimens. A third approach targets antibody production rather than antibody itself. Anti-CD20 therapy alone is insufficient, because the long-lived plasma cells that sustain anti-AAV IgG do not express CD20; combination regimens are therefore required. Rituximab with rapamycin has reduced anti-capsid antibody titers in a non-human primate model of AAV gene transfer (Mingozzi et al., 2012) and has been used clinically to control an anti-capsid response in an individual patient (Corti et al., 2014), while the addition of the proteasome inhibitor bortezomib to deplete plasma cells produced more effective reduction of pre-existing antibodies than B-cell depletion alone (Velazquez et al., 2017); analogous logic underpins B-cell depletion strategies designed to erase memory responses and facilitate tolerance induction (Biswas et al., 2020). More recently, a transient B-cell-focused regimen combining anti-CD20-mediated B-cell depletion with prolonged BAFF blockade prevented sustained anti-capsid neutralizing-antibody formation and enabled effective AAV8 re-administration after immune reconstitution in mice (Rana et al., 2024). Initiating immunosuppression before vector administration and extending BAFF blockade were important for suppressing both capsid- and transgene-directed adaptive responses. Although this study involved liver-directed AAV delivery rather than body-wide muscle transduction, it provides mechanistic evidence that coordinated targeting of B cells and their survival signals may be more effective for enabling redosing than CD20 depletion alone. Direct evidence relevant to DMD has also emerged. In mdx mice and mice pre-immunized with empty AAV9 capsids, an immunosuppressive regimen reduced anti-AAV antibody levels, increased AAV9-UFμDys1-mediated microdystrophin expression in skeletal and cardiac muscle, and allowed repeat administration to produce additional transgene expression that was not observed without immunosuppression (Saha et al., 2026). These findings extend the redosing concept from liver-directed models to a DMD-specific systemic microdystrophin setting. Nevertheless, the efficacy and safety of such protocols remain unproven in patients, particularly at the high vector doses required for body-wide muscle transduction. The purpose of such regimens is twofold: to lower pre-existing titers, and to blunt the post-dose anamnestic response that otherwise forecloses redosing. The trade-off remains substantial in this population. Patients with DMD are typically children already receiving chronic corticosteroids, and adding prolonged B-cell or plasma-cell depletion may increase the risks of infection, impaired vaccine responses, and delayed immune reconstitution. Although recent preclinical studies provide direct proof of concept for AAV redosing, the clinical risk–benefit profile of these regimens remains uncertain.

Tolerogenic co-administration. Rather than removing antibody after it has formed, tolerogenic platforms attempt to prevent its formation. Rapamycin-loaded synthetic vaccine particles (SVP-Rapamycin/ImmTOR), co-administered with vector, induce antigen-specific tolerance to the capsid, suppress anti-AAV IgG, and have enabled successful vector re-administration in mice and non-human primates (Meliani et al., 2018; Kishimoto, 2020; Ilyinskii et al., 2021). Conceptually this is the most attractive of the available strategies, because it is prophylactic rather than remedial and preserves general immune competence. It is also the least tested at the antigen loads relevant to DMD: tolerance induction that succeeds at liver-directed doses may behave differently when capsid protein is delivered at the quantities required for body-wide muscle transduction.

Serotype switching and antibody-evading capsids. Finally, the antigen can be changed rather than the host. Sequential administration of serologically distinct capsids has permitted repeated hepatic delivery in mice and non-human primates where cross-neutralization is limited (Majowicz et al., 2017), although the extensive cross-reactivity among natural AAV serotypes constrains how far this can be taken. More promising in the longer term is capsid engineering that simultaneously evades pre-existing antibodies and increases myotropism, since a vector that reaches therapeutic transduction at a lower dose reduces the antigenic burden that these mitigation strategies are otherwise required to counteract (El Andari et al., 2022; Shen et al., 2022; Vu Hong et al., 2024). Considered this way, improved capsids are not only a delivery advance but an immunological one, and lower-dose myotropic vectors and antibody-management protocols are best understood as complementary rather than alternative solutions. The logical endpoint of this reasoning is to remove the capsid altogether. Non-viral carriers elicit no anti-capsid neutralising response and are in principle repeatable without any of the mitigation strategies described above; whether they can deliver a therapeutically meaningful payload to the whole striated-muscle compartment is a separate and currently unresolved question, considered in Section 10.5.

What this does and does not resolve. Three qualifications are important, because the literature in this area is easily over-read. First, most of the supporting evidence derives from liver-directed protocols at vector doses well below those used for systemic microdystrophin delivery; extrapolation to DMD is plausible but not demonstrated. Second, and more fundamentally, all of these strategies address humoral anti-capsid immunity. They do not address the innate and complement-mediated axis that produced thrombotic microangiopathy, they do not address hepatotoxicity, and they do not address transgene-directed myositis, which is determined by the patient’s deletion boundaries rather than by serological status and would, if anything, be encountered more often if eligibility were broadened. Third, enabling redosing raises a question the field has not yet had to answer: whether repeated high-dose systemic AAV exposure is cumulatively tolerable, or whether each administration adds hepatic and innate immune burden that a growing child can absorb only a limited number of times.

The defensible conclusion is therefore intermediate. The single-use, seronegative-only character of current AAV therapy should no longer be described as an intrinsic property of the platform; it is an active research target, and partial solutions exist. But it has not been solved, and the strategies that would relax it carry costs of their own. On this view, safety and efficacy are not fully separable in DMD gene therapy: a therapy that delivers more dystrophin at the cost of severe hepatic, complement-mediated, or inflammatory muscle injury is not more effective in any meaningful sense. The most useful measure of progress may be the ratio between corrected muscle mass and total immunological burden—a ratio that improved capsids, lower doses, and rational immune management could each move in the same favorable direction.

## 10 Under-discussed constraints: cardiac coverage, satellite cells, one-time dosing, and manufacturing

Public discussion tends to focus on dystrophin expression, trial endpoints, regulatory decisions, and safety events. Several less visible constraints may nonetheless have a large influence on long-term outcomes.

### 10.1 Cardiac coverage

The heart is arguably the most important of these. Because skeletal muscle is accessible, biopsy data have understandably dominated molecular analysis, but the heart is both clinically decisive and difficult to observe directly (McNally et al., 2015). Since cardiac tissue is rarely sampled, myocardial transduction is usually inferred, and no DMD therapy can be considered comprehensive if it leaves cardiomyopathy largely unaddressed (Kamdar and Garry, 2016). Different AAV serotypes and promoters show distinct tropism, and some preclinical studies report substantial cardiac expression (Schröder et al., 2023); whether these levels are consistently reproduced in patients is less clear (Hart et al., 2024).

The absence of direct sampling does not, however, leave the heart unmeasurable, and it would be inaccurate to describe cardiac efficacy as inherently unobservable. A mature set of non-invasive surrogates is already deployed in DMD natural-history studies and interventional trials. Late gadolinium enhancement identifies replacement fibrosis and follows a characteristic inferolateral-to-global progression with age and declining systolic function (Hor et al., 2013; Silva et al., 2017). Native T1 mapping and ECV quantification detect diffuse interstitial expansion before ejection fraction falls and stratify disease severity in patients whose conventional indices remain normal (Olivieri et al., 2016; Soslow et al., 2016), while T2 mapping distinguishes active myocardial edema from established scar. Myocardial deformation imaging—CMR tagging or feature tracking, and speckle-tracking echocardiography—detects contractile impairment earlier and more reproducibly than ejection fraction, and serial circumferential strain has been used as a primary endpoint in a randomised DMD cardiac trial (Hagenbuch et al., 2010; Raman et al., 2015; Amedro et al., 2019). Circulating hs-cTnI adds a low-cost, frequently repeatable marker of ongoing myocyte injury that correlates with late gadolinium enhancement and native T1 and can identify episodes of acute injury between imaging timepoints (Voleti et al., 2020).

Recognizing these tools sharpens rather than dissolves the problem, for three reasons. First, they measure myocardial tissue state, not vector biodistribution or transgene expression; a stable ECV is compatible both with successful cardiac transduction and with a slowly progressing myocardium that has simply not yet declined. Inference from tissue state to delivery therefore remains indirect. Second, their temporal resolution is poorly matched to trial duration: fibrosis indices and strain change over years, so a 52-week study is unlikely to demonstrate cardiac benefit even where it exists, and equally unlikely to exclude it. Third, they carry practical and technical constraints—T1 and ECV values are sequence-, field-strength- and vendor-dependent and require standardized acquisition and local reference ranges (Messroghli et al., 2017); younger boys may require sedation; contrast administration raises additional considerations; and non-ambulatory patients with contractures and respiratory compromise tolerate prolonged scanning poorly, which is precisely the group in which cardiac endpoints matter most.

The appropriate conclusion is therefore not that cardiac efficacy cannot be assessed, but that it has been assessed less systematically and over shorter horizons than its clinical importance warrants. Future programs would be strengthened by pre-specifying harmonized CMR protocols with central reading, by collecting hs-cTnI at defined intervals for both safety and efficacy purposes, and by committing to cardiac follow-up windows of several years, so that myocardial imaging and biomarkers function as primary evidence streams rather than as exploratory addenda reported after the functional readouts have already determined how a trial is interpreted.

It is worth noting that the highest cardiac restoration reported for any dystrophin-directed AAV approach comes from a preclinical U7 study in which the heart could be assayed directly—a value that, in a patient, would be unverifiable by any currently available method.

### 10.2 The central nervous system: a compartment excluded by design

If the heart is under-observed, the brain has until recently been largely outside the therapeutic frame altogether. This is not because the neurological burden is small—cognitive and behavioral involvement affects a large minority of patients and is a major determinant of educational attainment, independence and quality of life (Section 2)—but because three distinct obstacles have made the CNS appear intractable, and because the field’s outcome measures have not required it to be addressed.

The first obstacle is access. Phosphorodiamidate morpholino oligomers do not meaningfully cross the blood–brain barrier, so the entire approved exon-skipping armamentarium acts on muscle alone. Tricyclo-DNA chemistry is a notable exception: it achieves partial exon skipping and restoration of brain dystrophin after systemic administration, with correction of behavioral abnormalities in dystrophin-deficient mice (Goyenvalle et al., 2015; Relizani et al., 2017), and intracerebroventricular oligonucleotide delivery likewise restores brain Dp427 and improves behavior (Zarrouki et al., 2022). For AAV, neonatal intravascular AAV9 crosses the immature barrier efficiently (Foust et al., 2009), and engineered variants transduce the adult CNS after systemic injection—though the best-characterised of these, AAV-PHP.B, depends on a receptor interaction restricted to particular mouse strains and does not translate to primates, a cautionary example of how readily CNS tropism data can be over-generalised (Deverman et al., 2016; Chan et al., 2017; Hordeaux et al., 2018; Goertsen et al., 2022).

The second obstacle is that the CNS has been actively excluded by construct design. Muscle-restricted promoters such as MHCK7, CK8e and Spc5-12 were adopted to concentrate expression in striated muscle and to minimize ectopic antigen presentation and immune activation (Section 4). That decision was defensible on immunological grounds, but its corollary is rarely stated: every microdystrophin product currently in clinical use or late-stage development is, by design, incapable of restoring dystrophin in neurons. The absence of a brain effect in these programs is therefore not an empirical finding about the difficulty of the CNS; it is a designed-in property. A related design question concerns the construct itself, since the C-terminal and syntrophin/dystrobrevin-binding regions omitted from many microdystrophins are precisely those that organise the neuronal dystrophin-associated complex at inhibitory synapses—so a construct optimised for sarcolemmal mechanics may not reconstitute the synaptic complex even if it reaches the brain. Two platform-specific inferences follow, and neither has been tested. AAV-U7 is not excluded by promoter design, since the cassette is transcribed from U7’s own natural promoter (Section 7.3); in exon 2 duplication genotypes, where the downstream internal promoters are intact and only Dp427 is lost, a vector reaching neurons would in principle restore full-length brain dystrophin. Conversely, transferrin receptor 1 is the canonical receptor for transcytosis across the blood–brain barrier, so TfR1-targeted conjugates are the one antisense platform with a plausible route into the CNS—although the affinity and valency requirements for transcytosis differ from those for muscle uptake, and the clinical constructs were not engineered for it.

The third obstacle is timing, and it cuts against the therapeutic case rather than for it. Unlike myofibre loss, the DMD brain phenotype is predominantly neurodevelopmental and largely static once established. This implies both that intervention would have to occur very early—plausibly earlier than current diagnostic pathways permit, which strengthens the argument for newborn screening (Section 11)—and that the achievable benefit may be prevention of divergence rather than reversal of an established deficit. Whether an already-established cognitive or behavioral phenotype in an older child is modifiable at all is unknown.

Recent preclinical work indicates that the first two obstacles are more tractable than has been assumed. In the mdx52 model, which lacks both Dp427 and Dp140 and reproduces the human loss pattern seen in most patients, a neuronal-targeted AAV microdystrophin restored brain dystrophin together with its interactors dystroglycan and syntrophin and the pre- and post-synaptic partners VGLUT1, gephyrin and GABA_A receptor, and improved emotional reactivity, anxiety-related behavior, cognition and obsessive-compulsive-like behavior; systemic administration, which achieved lower but far more widespread transduction including hindbrain and cerebellum, outperformed intracerebroventricular delivery, which produced higher forebrain transduction but a narrower behavioral benefit (Tetorou et al., 2026) (preprint). Two features of this result are relevant beyond the CNS. First, it is an unusually direct demonstration that breadth of distribution can matter more than local expression level—the same principle argued in Section 3 for skeletal muscle—since the route producing less forebrain transduction produced the better functional outcome. Second, and more provocatively, the same systemic neuronal-targeted vector expressed microdystrophin at neuromuscular junctions, corrected post-synaptic electrophysiological dysfunction, and normalised grip strength. If confirmed, this implies that a functional readout routinely interpreted as a measure of muscle contractility has a neural and junctional component, and that the strict partition between “muscle” and “brain” endpoints in DMD may be less clean than assumed. These findings are preclinical and, at the time of writing, not yet peer-reviewed, and the neonatal mouse is a permissive setting for CNS transduction in a way that a diagnosed human child is not; they should be read as establishing feasibility and motivating measurement rather than as evidence of clinical benefit.

Two costs must be set against this. Reaching the CNS by the systemic route requires either high vector doses, with the hepatic, complement and innate consequences described in Section 8, or direct CNS administration, which introduces its own procedural risk and, as the comparison above suggests, may not distribute adequately. And expressing a transgene in a new compartment expands the antigenic surface of the therapy, in an immunologically distinctive tissue, in patients who may be untolerised to the encoded epitopes (Section 8.2). A brain-directed strategy is therefore not simply an addition to a muscle-directed one; it changes the risk calculus.

The immediate practical implication is nonetheless modest and achievable. Neurocognitive and neurobehavioral outcomes are almost never collected systematically in DMD gene therapy trials, even as exploratory measures, with the consequence that the field has no dataset with which to determine whether existing therapies exert any CNS effect, or whether high-dose systemic AAV affects the brain adversely. Given that validated instruments exist and impose little burden, their omission is difficult to justify. Until such data are collected, statements that DMD gene therapy does not address the brain will remain accurate but uninformative, since the question has not been asked.

### 10.3 Satellite cells and durability

The regenerative compartment is a second constraint. Long-lasting episomal expression in post-mitotic cells is a genuine strength of AAV, but dystrophic muscle is not static; satellite cells are repeatedly recruited to repair damaged fibers, and if they remain uncorrected, newly regenerated tissue may dilute the therapeutic effect over time (Kwon et al., 2020; Mollard et al., 2022). This concern applies both to gene replacement and to AAV-delivered splice-modulating constructs. As the role of satellite cells in DMD has become better understood, it has become clearer that a therapy stabilizing mature fibers while leaving the regenerative compartment genetically uncorrected may reduce the rate of functional decline while leaving the cycle of degeneration and imperfect regeneration intact, so that the corrected fraction of muscle falls progressively as uncorrected satellite cells contribute new myonuclei (Morgan and Muntoni, 2021; Kodippili and Rudnicki, 2023). This is one reason genome editing continues to attract interest: if editing could be delivered efficiently to satellite cells as well as to myonuclei, the corrected state might become self-renewing rather than progressively diluted. Current delivery platforms, however, have not yet solved the problem of safely and specifically targeting this compartment at scale.

### 10.4 The consequences of one-time treatment

The difficulty of redosing AAV-based therapies is more than a practical inconvenience in DMD. Because treatment is effectively single-use, each infusion carries considerable strategic weight, and the timing of treatment becomes a critical and largely irreversible decision. Treating early leaves durability uncertain in a still-growing body; treating later means that fibrosis, cardiomyopathy, and immune vulnerability may reduce benefit or increase risk. This is a biological tension inherent in the platform rather than a simple scheduling matter. Strategies to enable treatment in seropositive patients, or to permit redosing—apheresis and immunoadsorption, enzymatic IgG cleavage, plasma-cell-directed immunosuppression, tolerogenic co-administration, and serotype switching (Section 8.3)—are therefore of considerable importance, since any advance that relaxes the single-use constraint would simultaneously widen access and convert the timing decision from an irreversible one into a revisable one (Potter et al., 2024; Oskoui et al., 2025). Whether that relaxation is achievable at the vector doses DMD requires remains the central open question. The logical endpoint of this reasoning is to remove the capsid altogether. Non-viral carriers elicit no anti-capsid neutralising response and are in principle repeatable without any of the mitigation strategies described above; whether they can deliver a therapeutically meaningful payload to the whole striated-muscle compartment is a separate and currently unresolved question, considered in Section 10.5.

### 10.5 Manufacturing as part of the therapeutic profile

Manufacturing deserves more attention than it typically receives. In DMD, production quality is not only a regulatory requirement but a determinant of the therapeutic profile. Vector genome integrity, potency, full-versus-empty capsid ratio, residual host-cell DNA, endotoxin burden, and batch-to-batch consistency directly affect effective dose, immunogenicity, and tissue transduction (El Andari et al., 2022). Empty capsids, for example, may increase antigenic burden without contributing benefit, so suboptimal preparations can amplify toxicity while reducing efficacy (Wright, 2014; Costa Verdera et al., 2020). This is particularly relevant when comparing programs, because apparent differences between constructs or serotypes may partly reflect manufacturing and formulation choices rather than promoter or capsid biology alone. Where doses already approach immunological limits, manufacturing variability is not incidental; it is a clinically relevant variable that should be reported and discussed more explicitly.

Dose reporting is a specific instance of this problem. Because vg/kg values depend on the quantitation platform—qPCR versus ddPCR, amplicon location within the cassette, whether the template is linearised, and how self-complementary genomes are counted—the same physical dose can be assigned materially different numerical values by different laboratories (Fagone et al., 2012; Werling et al., 2015; Dobnik et al., 2019; Furuta-Hanawa et al., 2019). Standardized reference materials were developed to address this but are not universally adopted (Ayuso et al., 2014; D’Costa et al., 2016). The consequence is that cross-program statements about dose-dependent hepatotoxicity or complement activation rest on a unit that is only approximately shared. Where safety thresholds are being compared across sponsors, the quantitation method should be reported alongside the dose, and, ideally, values should be traceable to a common reference standard. Throughout this review, doses cited from different programs should be read with this qualification in mind. This difficulty is compounded by the fact that the doses under comparison were not independently derived. Because the 1–2 × 10^14^ vg/kg envelope entered DMD from the SMA programme, and because the DMD-specific dose-escalation studies were themselves reported in the same assay-dependent units (Section 4.1), a numerical value of uncertain physical meaning became a *de facto* cross-sponsor standard before it had been re-derived by any single harmonised method.

Taken together, these constraints suggest that expression in a skeletal muscle biopsy, while important, is no longer a sufficient measure of progress. The more decisive questions are whether a therapy protects the heart, whether it influences or bypasses regenerative turnover, whether its effect can persist without redosing, and whether it can be manufactured consistently enough to remain reproducible outside specialized trial settings.

## 11 A convergent rather than competitive future

The future of DMD therapy is often presented as a contest among platforms: exon skipping versus gene replacement, microdystrophin versus full-length dystrophin, AAV versus non-viral delivery, gene transfer versus editing. This framing is convenient but misleading. The main strategies under development are better understood as attempts to solve different parts of the same multivariable problem, which points toward convergence rather than simple replacement.

### 11.1 Improved capsids

Engineered myotropic AAV variants may be among the more immediately relevant developments, because they address delivery directly. Capsids that achieve higher muscle transduction with lower liver uptake and lower total dose could, in principle, raise muscle transduction while lowering total vector dose, and thus improve efficacy and reduce hepatic and complement-mediated burden simultaneously rather than exchanging one for the other—provided the improved tropism is reproduced in patients and not only in inbred rodents (El Andari et al., 2022; Shen et al., 2022; Vu Hong et al., 2024). Early clinical reports are encouraging, but the key question is whether improved biodistribution is maintained across diverse patients rather than only in small early cohorts. If validated, better vectors could also change which cargo is worth delivering, making a more biologically complete but previously dose-limited construct feasible.

### 11.2 Larger dystrophins

This leads to a second direction: larger therapeutic proteins. Microdystrophin was an effective response to the single-vector constraint, but it is not the endpoint of protein design. Multi-vector approaches based on trans-splicing, homologous recombination, ribozyme-mediated RNA ligation, and particularly split intein–mediated protein trans-splicing have renewed interest in delivering larger mini-/midi-dystrophins or full-length dystrophin (Lindley et al., 2024; Tasfaout et al., 2024; Zhou et al., 2024). Earlier versions were limited by low efficiency and unpredictable reconstitution; more recent work has improved domain design, split-site selection, vector stoichiometry, promoter choice, and capsid pairing (Zhou et al., 2024; Tasfaout et al., 2025). The rationale is functional rather than aesthetic: larger dystrophins may better preserve properties compromised by aggressive truncation, including aspects of membrane organization and cardiac function that could matter over longer follow-up (Zhou et al., 2024; Tasfaout et al., 2025). The principal challenge is whether multi-vector delivery can be made sufficiently reliable in humans, given the need for co-transduction of the same cell and the added manufacturing and immunological complexity.

### 11.3 More capable splice modulation

A third direction is more sophisticated splice modulation. Conventional PMO-based exon skipping has shown that endogenous reframing can work, generally with modest efficiency (Mendell et al., 2013). Peptide conjugates, antibody-linked platforms, and AAV-U7 systems represent efforts to increase the depth, breadth and tissue distribution of splice correction ((Betts et al., 2012; Sugo et al., 2016) Section 7.2), and in the U7 case, for permissive genotypes, to change the nature of the product from a truncated to a full-length protein, and multi-exon skipping is attractive because it could extend applicability to a larger proportion of *DMD* mutations (Aoki et al., 2012; Roberts et al., 2023). Unlike microdystrophin, splice modulation preserves endogenous regulation and avoids introducing a large exogenous protein. Its limitations are that it typically restores limited amounts of truncated dystrophin and, unless encoded by a durable vector, generally requires repeated administration (Hanson et al., 2021). In a precision-medicine context, this is not necessarily a disadvantage: for some patients, repeatable, lower-risk endogenous correction may be preferable to a single high-burden infusion.

### 11.4 Editing aimed at durability

A fourth direction is genome editing. CRISPR-based exon excision, splice-site disruption, base editing, and prime editing have all shown preclinical potential. Editing is distinctive in offering native-locus correction and, at least in principle, durable benefit if satellite cells are reached. It nonetheless inherits the delivery problem and adds concerns about off-target effects, large deletions, chromosomal rearrangements, and immune responses to editing enzymes (Monteys et al., 2021; Li et al., 2023). Progress is likely to depend on several advances occurring together: smaller and more precise editors, transient expression systems, muscle- or satellite-cell-specific regulatory elements, improved capsids to reduce the vector burden required for *in vivo* editing, and—of particular importance given that AAV genome integration at nuclease-induced breaks is a liability specific to viral delivery of editors—non-viral or hybrid delivery platforms, which are considered separately in Section 10.5. The initiation of first-in-human AAV-CRISPR trials in DMD illustrates both the promise and the added complexity of this direction, since integration events at cut sites represent a genomic safety liability distinct from those already characterized for episomal AAV cargoes. Editing is therefore not a solution waiting immediately beyond current therapies, but a demanding extension of the field’s broader shift toward durability and biological completeness.

### 11.5 Non-viral delivery: decoupling the durability of the effect from the persistence of the vector

A fifth direction follows directly from the argument of Section 8: if anti-capsid immunity is the principal reason that AAV administration is effectively single-use and restricted to seronegative patients, then delivery systems that do not present a viral capsid are not merely an alternative platform but a structural response to that constraint. Lipid nanoparticles (LNPs), polymeric carriers, and inorganic nanoparticles share several properties that are attractive in DMD specifically. They provoke no anti-capsid neutralising antibody response and are therefore, in principle, repeatable; they impose no fixed packaging limit, so cargo size is governed by formulation stability rather than by a 4.7-kb ceiling; and they do not deliver a persistent DNA genome, which removes both the risk of vector integration at nuclease-induced double-strand breaks discussed above and the concern that an episomal cassette will be diluted by regenerative turnover (Hou et al., 2021).

The last of these points is worth stating precisely, because it inverts the durability logic that governs AAV. For protein replacement, transient delivery is a liability: dystrophin must be present continuously at the sarcolemma, and an LNP-delivered mRNA encoding even a full-length dystrophin would produce protein for days, not years. For genome editing, transient delivery is an asset. A ribonucleoprotein (RNP) or mRNA-encoded editor need only be present long enough to make a permanent change at the endogenous locus, after which its clearance is desirable rather than problematic, since it limits off-target activity and reduces exposure to a bacterial nuclease that is itself immunogenic. Non-viral delivery therefore separates the durability of the therapeutic effect from the persistence of the delivery agent—precisely the dissociation that episomal AAV cannot achieve. That this is now clinically feasible was established outside DMD by LNP-delivered CRISPR-Cas9 targeting hepatic transthyretin, which produced durable, dose-dependent protein reduction in patients (Gillmore et al., 2021).

The obstacle is tropism. Conventional ionisable LNPs distribute overwhelmingly to the liver through apolipoprotein E adsorption and hepatocyte LDL-receptor uptake, and skeletal muscle and myocardium are poorly transfected after systemic administration (Dilliard et al., 2021; Hou et al., 2021). Several strategies are being pursued to redirect them. Selective organ targeting (SORT), in which a supplemental charged lipid shifts the protein corona and hence the destination organ, has achieved tissue-specific mRNA delivery and Cas-mediated editing in lung, spleen and liver, demonstrating that tropism is formulation-tunable rather than fixed (Cheng et al., 2020; Dilliard et al., 2021); high-throughput *in vivo* barcoded screening has been used to identify formulations with non-hepatic tropism (Sago et al., 2018). Directly relevant to muscle, an LNP formulation with low immunogenicity permitted repeated intramuscular administration of Cas9 mRNA in mice with cumulative editing across doses—an explicit demonstration of the redosing advantage that AAV cannot currently match (Kenjo et al., 2021)—while systemically administered nanoparticles carrying Cas9 RNP have achieved tissue-selective editing *in vivo* (Wei et al., 2020). Non-lipid platforms have also been applied to DMD itself: gold-nanoparticle-templated Cas9 RNP with donor DNA (CRISPR-Gold) corrected the dystrophin locus and improved muscle strength after intramuscular injection in mdx mice with minimal off-target activity (Lee et al., 2017), and biodegradable polymer nanocapsules have delivered Cas9 RNP *in vivo*, including to muscle (Chen et al., 2019). The heart is more difficult still; lipidoid nanoparticles carrying modified mRNA transfect myocardium after direct intramyocardial injection, but efficient, non-invasive, systemically administered cardiac delivery remains unsolved (Turnbull et al., 2016; Magadum et al., 2019).

Three limitations temper the enthusiasm, and the first is quantitative. Almost all muscle-directed non-viral successes to date have involved local injection or small rodents; DMD requires transfection of a tissue compartment amounting to roughly a third of body mass, and the potency per dose currently achievable in muscle is orders of magnitude below what body-wide correction would demand. Second, non-viral does not mean non-immunogenic. Ionisable lipids are intrinsically inflammatory and possess adjuvant activity (Alameh et al., 2021; Ndeupen et al., 2021), and PEGylated components elicit anti-PEG antibodies that mediate accelerated blood clearance and complement-activation-related pseudoallergy, with titers boosted by prior mRNA vaccine exposure (Kozma et al., 2020; Ju et al., 2022). Repeat dosing is therefore easier than with AAV but not unconstrained, and the innate and complement-mediated axis identified in Section 8.1 does not disappear simply because the capsid does. Third, editors delivered as mRNA or RNP are transient by design, so any cell not edited during the exposure window—including quiescent satellite cells—remains uncorrected, and re-administration becomes a requirement rather than an option.

A reasonable near-term expectation is therefore not that non-viral delivery will displace AAV for dystrophin replacement, for which continuous protein expression is required and transient cargo is poorly suited, but that it will be adopted first where its properties are advantageous: as a redosable vehicle for genome editors, as a means of reaching the regenerative compartment repeatedly rather than once (Section 9.2), and as a route to treating patients excluded by anti-AAV seropositivity. In that role it is complementary to the strategies discussed above rather than competitive with them, and it is the clearest available answer to the question of what a genuinely repeatable dystrophin-directed therapy might eventually look like.

### 11.6 Combination rather than succession

These directions point toward a combinatorial rather than sequential future. A young ambulatory patient with a favorable immune profile might be a candidate for a highly myotropic AAV construct, whereas a seropositive patient with a skip-amenable mutation might be better served by an advanced exon-skipping approach. Patients treated first with one-time gene transfer might later require adjunctive strategies directed at satellite-cell correction, anti-fibrotic remodeling, cardiomyopathy, or—if brain-directed approaches mature and can be deployed early enough—the neurological compartment, which no muscle-restricted construct will reach. Agents such as givinostat and vamorolone, which do not restore dystrophin directly, may nonetheless improve the tissue substrate into which gene- or editing-based therapies are introduced, or reduce the corticosteroid burden that a treated child carries for decades ((Bettica et al., 2016; Dang et al., 2024; Mercuri et al., 2024) Section 8). Because no trial has yet been designed to test sequence or interaction between a substrate-directed agent and a dystrophin-restoring one, the most clinically consequential combination in this field remains entirely unevaluated. It is therefore unlikely that a single modality will dominate; the more probable outcome is a set of strategies matched to biological need and disease stage.

## 12 Patient selection, trial design, and newborn screening

As the molecular tools improve, the clinical and regulatory environment required to evaluate them becomes more demanding—not because standards are unreasonable, but because DMD combines urgent unmet need, modest patient numbers, developmental variability, heterogeneous progression, and therapies that may act as gradual disease modifiers rather than producing rapid functional gains (Fischer et al., 2024; Johnson et al., 2025). Three decisions determine whether such a therapy can be evaluated at all—who is treated, at what dose, and against what measured over what interval—and each has been made in this field with less explicit justification than the molecular design it is intended to test.

### 12.1 Patient and dose selection: stratification by biology rather than by convenience

Patient selection is a central issue. Age, ambulatory status, mutation class, fibrosis burden, cardiac involvement, steroid history, and serological status all influence both efficacy and risk (Goedeker et al., 2023; Verma et al., 2023). Many earlier trial designs treated DMD cohorts as more homogeneous than they are, which may have diluted therapeutic signals: a therapy most effective in younger ambulatory patients, before substantial fibrosis, could show only a weak average effect when evaluated across a broad age range. Conversely, restricting development to younger children risks leaving older patients without options and may overstate benefit if the trial population is unusually amenable to treatment. Future studies would benefit from biologically coherent stratification rather than broad inclusion. Dose selection deserves the same scrutiny as patient selection, and has received less. Because the effective dose in DMD was established by ascending dose-finding in mdx mice within an envelope inherited from SMA, and then confirmed clinically at the top of that envelope rather than titrated downwards in patients (Section 4.1), the field has substantial safety data at the top of the range and very little clinical efficacy data below it. Prospective evaluation of lower doses—whether alone, as in the GNT0004 approach, or in combination with myotropic capsids that achieve therapeutic transduction at reduced vector load (Section 10.1) — is therefore not merely a means of reducing toxicity but a way of establishing, for the first time in this indication, where the lower boundary of the therapeutic window actually lies.

### 12.2 Objective criteria for revising primary and secondary endpoints

It is easy to assert that DMD endpoints are inadequate and harder to specify what would count as an improvement. We therefore set out explicit criteria, an operational threshold for each, and the consequence when a criterion is unmet (Table 5), together with an appraisal of the endpoints currently in use (Table 6). The criteria are not novel in themselves; what is lacking in this field is their systematic application before rather than after a pivotal trial.

**TABLE 5:** Objective criteria for accepting, revising or rejecting a primary endpoint in a DMD disease-modifying trial.

#	Criterion	Operational metric	Proposed threshold	Consequence if unmet
C1	Mechanistic alignment	Endpoint lies causally downstream of the stated mechanism in the treated compartment	Explicit causal pathway pre-specified	Re-site endpoint to the compartment actually targeted
C2	Measurement reliability	Test–retest ICC; minimal detectable change at 95% confidence (MDC_95_) in the same age band and ambulatory stratum	ICC ≥0.85; MDC_95_ reported, not assumed	Endpoint unusable as primary; use as supportive
C3	Detectability within the planned window	t_detect = MDC_95_ ÷ (expected between-arm annual difference); and n/arm ≈16σ^2^/Δ^2^	t_detect ≤ planned follow-up; feasible n	Extend follow-up or change endpoint — do not increase α
C4	Freedom from developmental confounding	Proportion of cohort <7 years; pre-specified age × treatment interaction; use of age-normalised scores	Stratified randomisation with pre-specified interaction test	Restrict age band or normalise to age-referenced z-scores
C5	Absence of floor/ceiling effects	Proportion at scale floor or ceiling at baseline and projected at end of study	<15% at either extreme	Substitute a stage-appropriate measure
C6	Scale properties	Ordinal vs. interval; Rasch/IRT fit statistics	Interval-level (Rasch-transformed) scores for summed scales; velocity (1/t) for timed tests	Transform before analysis, pre-specified
C7	Estimand correctly specified for a decline-modifying effect	ICH E9(R1) estimand; absolute change vs. slope vs. time-to-event	Slope-based or time-to-event primary when the expected effect is slowed decline	Absolute-change endpoint is mis-specified and will under-detect
C8	Handling of intercurrent events	Loss of ambulation, scoliosis surgery, ventilation treated as intercurrent events, not missing data	Pre-specified estimand strategy (e.g., composite/while-alive)	Bias of unknown direction
C9	Established clinical meaningfulness	Anchor-based MCID in the same population and stratum	MCID available and exceeded by the target Δ	Effect uninterpretable even if statistically significant
C10	Compartment coverage	Whether ambulatory, respiratory, cardiac and cognitive domains are represented	Cardiac and respiratory measures pre-specified with allocated α	Trial cannot support a claim of systemic benefit

ICC, intraclass correlation coefficient; IRT, item response theory; MCID, minimal clinically important difference.

**TABLE 6:** Appraisal of endpoints currently used in DMD trials against the criteria in Table 5.

Endpoint	Fails	Recommended revision	Proposed tier
NSAA total score, change at 52 weeks	C3, C4, C6, C7	Rasch-transform to interval scale; analyse as slope over ≥104 weeks with ≥5 post-baseline assessments; stratify at 7 years with pre-specified interaction	Primary (as slope)
Time to rise from supine (seconds)	C5, C6, C7	Analyse as velocity (1/s); pre-specify handling of inability to perform as an intercurrent event, not imputation	Key secondary
10-m walk/run	C5, C6	Velocity transformation; not informative near loss of ambulation	Key secondary
6-min walk distance	C4, C9 partly	Retain only in ≥7-year ambulatory cohorts; age/height-normalised	Secondary
Four-stair climb	C5, C6	Velocity transformation	Secondary
PUL 2.0	C9	Retain for non-ambulatory cohorts, where it escapes C5	Primary in non-ambulatory trials
FVC % predicted	C3	Slope over ≥104 weeks; principal endpoint after loss of ambulation	Key secondary/primary in late-stage trials
Stride velocity 95th centile (SV95C, wearable)	—	Already qualified by EMA as a valid secondary endpoint in ambulant DMD; continuous home measurement raises reliability and removes effort/motivation confounding	Key secondary, strongly recommended
Time to loss of ambulation	C3 (duration)	Unambiguous, patient-relevant, free of C4 and C6; requires ≥3 years or pooled/registry-linked follow-up	Key secondary; primary in long trials
CMR ECV/native T1/LGE/circumferential strain	C3	Harmonised acquisition, central blinded reading, ≥156-week window with allocated α	Key secondary with α allocation
hs-cTnI	—	Defined-interval sampling; serves both safety and efficacy	Safety + supportive
Dystrophin (Western blot/IF)	C9, and fails surrogate validation (see B-4)	Retain as mechanistic/supportive tier only, with single pre-specified assay, reported LLOQ and inter-laboratory CV, and pre-specified mediation analysis	Mechanistic, not primary
Neurocognitive/behavioural instruments	Currently absent	Add as exploratory at minimal cost (Section 10.2)	Exploratory

The decisive criterion is detectability, and it can be computed rather than debated. For an endpoint with minimal detectable change MDC_95_ and an expected between-arm difference Δ accruing at a constant annual rate, the time required for the effect to exceed measurement noise is t_detect = MDC_95_ ÷ Δ_annual, and the sample size required for 80% power at two-sided α = 0.05 is approximately n per arm ≈16σ^2^/Δ^2^, where σ is the standard deviation of change over the trial window. Applying this to the endpoint on which several pivotal DMD trials have turned is illuminating. Taking an annual NSAA decline of approximately 3 points in ambulatory boys aged 7–10 years, an MDC_95_ in the region of 4 points, and a therapy that slows decline by 40%, the expected between-arm difference is about 1.2 points at 52 weeks, 2.4 points at 104 weeks and 3.6 points at 156 weeks. With a standard deviation of 52-week change of approximately 4.5 points, detecting the 52-week difference requires on the order of 225 participants per arm; at 104 weeks, with a standard deviation of change of approximately 6 points, the requirement falls to approximately 100 per arm. Pivotal DMD trials have generally enrolled fewer than 70 per arm and used a 48- to 52-week primary endpoint. On these assumptions, a therapy producing a genuine 40% slowing of decline would be expected to miss such an endpoint most of the time. The specific figures must be re-derived from contemporary natural-history datasets and will differ by age band and steroid regimen, but the structure of the calculation does not depend on them, and it converts the recurring dispute over whether these trials were “negative” into a question about design adequacy that can be settled arithmetically before enrolment. It follows that when criterion C3 is unmet, the correct response is to lengthen follow-up or change the endpoint, not to enlarge the secondary endpoint family or to rely on *post hoc* subgroups—the route that ataluren has already shown to be unreliable (Section 8.3).

Four specific revisions follow, and each is implementable now. First, the estimand should match the expected effect: for a therapy that slows decline rather than producing gains, the primary quantity should be a slope estimated from at least five post-baseline assessments over at least 104 weeks, or a time-to-event outcome, rather than absolute change at a single visit (criterion C7). Second, scale properties should be corrected before analysis: summed ordinal scales such as NSAA should be Rasch-transformed to interval level, and timed tests should be analysed as velocities (1/time) rather than raw seconds, which reduces skew, mitigates floor effects and preserves information from slower performers (criterion C6). Third, developmental confounding should be handled by design: because motor function in boys under approximately 7 years is still maturing, age should be a stratification factor with a pre-specified age × treatment interaction test, or scores should be expressed relative to age-referenced norms (criterion C4; Mercuri et al., 2016). Fourth, the endpoint family should span the compartments in which the disease actually causes harm (criterion C10). This means pre-specifying respiratory and cardiac endpoints with formally allocated α—CMR extracellular volume, native T1 and circumferential strain with harmonised acquisition and central blinded reading, over a window of at least 3 years, because these indices evolve over years and are otherwise structurally disadvantaged by 1-year primary endpoints (Section 10.1) — and adding neurocognitive and behavioural instruments at least as exploratory measures (Section 10.2).

Two further instruments deserve explicit endorsement. Continuous wearable measurement of the stride velocity 95th centile has been qualified by the European Medicines Agency as a valid secondary endpoint in ambulant DMD; because it is measured at home over weeks rather than in a single supervised session, it improves reliability, removes effort and motivation as confounders, and is one of very few measures likely to register the exercise-dependent limitations predicted for microdystrophins lacking the nNOS-anchoring repeats (Section 3.2). And where several partially independent domains each move modestly—the pattern most consistent with the trial record—a hierarchical composite analysed by a win-ratio or Finkelstein–Schoenfeld approach, ordering death, loss of ambulation, loss of a defined motor milestone, functional slope and respiratory slope, is statistically more efficient than testing each separately and is closer to how clinicians and families weigh outcomes (Finkelstein and Schoenfeld, 1999).

### 12.3 Objective criteria for the use of molecular endpoints and external controls

Because accelerated approvals in DMD have rested on dystrophin quantification, the criteria a molecular measure must satisfy to support such a claim should be stated explicitly. We propose five, adapted from the standard operational definition of a surrogate (Prentice, 1989): (i) a single pre-specified assay with reported lower limit of quantification and inter-laboratory coefficient of variation below 20%; (ii) a defined normalisation reference and muscle-content adjustment, reported rather than assumed; (iii) a demonstrated exposure–or dose–response relationship; (iv) a pre-specified mediation analysis showing that a substantial proportion—we suggest at least half—of the treatment effect on the clinical endpoint is explained by the molecular change; and (v) consistency of the molecular–clinical association across independent trials, ideally by meta-regression. Current dystrophin measurements satisfy the first three partially at best and have not been tested against the last two. The appropriate conclusion is not that molecular endpoints should be abandoned, but that they belong to a mechanistic tier: they can license conditional access and they can falsify a mechanism, but they cannot at present substitute for functional evidence, and treating them as though they can is what generated the interpretive impasse described in Section 6.

Similar explicitness is needed for external controls, which will remain necessary in this population. We suggest four minimum requirements: pre-specified matching variables and analysis plan, registered before data access; demonstrated covariate overlap after matching, with standardised mean differences below 0.1 on prognostic variables; at least one negative-control outcome that treatment should not affect; and quantitative bias analysis reporting the magnitude of unmeasured confounding that would be required to nullify the observed effect. External comparators meeting these conditions are informative; those assembled after a concurrent comparison has proved unfavourable are not, and the distinction should be drawn on documentation rather than on plausibility.

Finally, the vamorolone programme illustrates a design option that the dystrophin-restoration field has largely ignored (Section 8.1). Where a therapy is expected to be comparable in efficacy but differentiated in safety or burden, a non-inferiority trial against an active comparator on a well-characterised functional endpoint is both achievable and interpretable, whereas a superiority trial against natural decline in a small cohort over 1 year frequently is not.

### 12.4 Newborn screening: preserving the opportunity to treat

These considerations connect to newborn screening. The earlier DMD is identified, the greater the opportunity to intervene before irreversible fibrosis, contractures, and cardiopulmonary decline reshape the therapeutic substrate (Kemper et al., 2025). Historically, newborn screening raised concerns because early diagnosis preceded clearly effective disease-modifying options; as gene transfer, exon skipping, and future editing platforms mature, the case for earlier identification strengthens. This shift has recently been enacted at the policy level. In December 2025, the U.S. Department of Health and Human Services formally added DMD to the Recommended Uniform Screening Panel (RUSP) following a period of public comment and an evidence-based review. Notably, the rulemaking record accompanying this decision explicitly acknowledged the interpretive tension emphasized throughout this review: several public comments noted the absence of data demonstrating that treatment initiated in the newborn period improves infant outcomes, and the evidence review concluded that although treatment may improve outcomes, further research is needed to establish optimal treatment timing. At the time of this decision, only a small number of U.S. states had operational DMD newborn screening programs, with several others having enacted but not yet implemented screening legislation, indicating that a substantial gap remains between panel-level recommendation and population-level screening access. This gap is itself an instance of the broader delivery-and-infrastructure constraint: earlier diagnosis creates the opportunity for earlier intervention only insofar as screening programs, treatment centers, and evidence on optimal treatment timing are simultaneously in place. From a translational standpoint, newborn screening is not only about diagnosis but about preserving the opportunity to treat while the tissue substrate remains favorable.

### 12.5 Infrastructure, cost, and operational accessibility

Finally, clinical translation cannot be separated from healthcare infrastructure and cost. Systemic gene therapies for DMD are logistically demanding, carry meaningful risk, and are expensive. They require centers capable of immune screening, peri-infusion management, hepatic and complement surveillance, long-term follow-up, and coordinated cardiac and pulmonary care. Some health systems may be unable to deliver such therapies safely even where they are approved, which creates a dual challenge: ensuring that approvals are scientifically justified, and ensuring that approval does not produce a therapy that is nominally available but operationally inaccessible. Progress in DMD will therefore depend on more than vector design—on earlier diagnosis, more intelligent stratification, endpoints matched to mechanism, and health systems organized to deliver and monitor these therapies responsibly.

Preliminary observations from the U7 exon 2 skipping trial, in which restoration efficiency appeared inversely related to age at dosing and which included the youngest patient yet treated with gene therapy for DMD (Roberts, 2023), are among the few clinical data bearing directly on this question.

## 13 Conclusion

DMD entered the genomic era with an apparently favorable premise: one locus, one missing protein, one clear therapeutic goal. Three decades of work indicate that this premise described only part of the problem. The locus is singular and the therapeutic objective shared, but the mutation spectrum is not—it shapes residual protein, isoform loss, cognitive involvement, eligibility and transgene immunogenicity—and neither is the treatment problem: protein design, body-wide delivery, cardiac coverage, immune recognition, regenerative turnover, disease stage, and trial architecture together determine whether molecular correction translates into clinical benefit.

A central lesson of the past decade is not that any single therapy has prevailed, but that the principal constraints are now clearer. AAV-microdystrophin demonstrated that systemic dystrophin expression is achievable and can be clinically relevant, while also showing that a feasible therapy may remain biologically incomplete. Exon skipping showed that even low-level endogenous restoration is not without value, while also showing that modest correction may not transform the disease; its AAV-U7 variant went further, demonstrating in a rare permissive genotype that full-length wild-type dystrophin can be restored in patient muscle, at a fraction of the conventional vector dose and without introducing an antigenic protein, and thereby isolating episomal persistence and genotype breadth as the residual constraints—while offering the field its only opportunity to test whether structural completeness matters clinically. Genome editing has clarified the path toward durability and native-locus repair while highlighting the unresolved challenges of delivery and safety. Non-viral delivery, in turn, offers the cleanest theoretical escape from capsid immunity and the single-use constraint, and uniquely decouples a permanent therapeutic effect from a persistent delivery vehicle, but has not yet demonstrated the potency required to transfect a tissue compartment of this size. Immune toxicities, once treated as regrettable complications, are now central design variables—as is dose itself, which entered DMD as an exposure envelope inherited from spinal muscular atrophy, was then refined within the disease by dose-escalation in mdx mice and confirmed in early clinical testing, but has still not been titrated downwards in patients and so remains undefined at its lower edge—and, increasingly, tractable ones, as antibody-depleting, enzymatic, plasma-cell-directed and tolerogenic strategies begin to convert seropositivity and single-use administration from fixed exclusions into manageable parameters; cardiac protection, once regarded as a downstream consideration, appears essential to any adequate definition of success; and satellite-cell biology, previously peripheral, is increasingly recognized as part of the durability problem. To these should be added the central nervous system, which current constructs exclude by design rather than by necessity, and which recent preclinical work suggests may be both accessible and functionally responsive—though on a developmental timescale that would require intervention earlier than present diagnostic pathways generally allow.

This supports a broader conclusion. The most successful interventions are unlikely to be those producing the highest local dystrophin value in a biopsy, but those—more probably, therapeutic architectures rather than single agents—that balance several requirements at once: adequate functional protein, sufficiently broad coverage of fibres, myonuclei and tissue compartments—correct localization being necessary but, as these platforms already demonstrate, not sufficient—meaningful cardiac effect, a tolerable immunological burden, sufficient persistence, and deployment early enough to act on repairable muscle. No current platform fully meets these criteria, though several are approaching them from different directions.

For these reasons, two interpretive tendencies are best avoided: treating any measurable dystrophin restoration as evidence that the core problem is solved, and treating every missed endpoint or safety event as evidence that a strategy is fundamentally flawed. Neither is well supported by the evidence, which is more consistent with progress that is real but conditional on delivery efficiency, disease stage at treatment, immunological status, and the endpoint used to measure it. The biology is well characterized, the translational obstacles are now identifiable, and further advances are likely to come from integrating what has already been learned rather than repeatedly rediscovering the same limits.
